# Design, Synthesis, and Antifungal Activity of *N*-(alkoxy)-Diphenyl Ether Carboxamide Derivates as Novel Succinate Dehydrogenase Inhibitors

**DOI:** 10.3390/molecules29010083

**Published:** 2023-12-22

**Authors:** Bo He, Yanhao Hu, Wang Chen, Xu He, Enpei Zhang, Mengxu Hu, Pu Zhang, Wei Yan, Yonghao Ye

**Affiliations:** 1 State & Local Joint Engineering Research Center of Green Pesticide Invention and Application, College of Plant Protection, Nanjing Agricultural University, Nanjing 210095, China; hebo@njau.edu.cn (B.H.); huyh@stu.njau.edu.cn (Y.H.); 2022102110@stu.njau.edu.cn (W.C.); 2022102112@stu.njau.edu.cn (X.H.); 12121413@stu.njau.edu.cn (E.Z.); 2021102127@stu.njau.edu.cn (M.H.); yanwei@njau.edu.cn (W.Y.); 2 Key Laboratory of Integrated Management of Crop Diseases and Pests, Ministry of Education, Nanjing 210095, China; 3 Research & Development Center, Jiangsu Flag Chemical Industry Co., Ltd., Nanjing 210095, China; zhangpu@flagchem.com

**Keywords:** succinate dehydrogenase (SDH), antifungal activity, fungicide, *N*-(alkoxy)diphenyl ether carboxamide

## Abstract

Succinate dehydrogenase (SDH, EC 1.3.5.1) is one of the most promising targets for fungicide development and has attracted great attention worldwide. However, existing commercial fungicides targeting SDH have led to the increasingly prominent problem of pathogen resistance, so it is necessary to develop new fungicides. Herein, we used a structure-based molecular design strategy to design and synthesize a series of novel SDHI fungicides containing an *N*-(alkoxy)diphenyl ether carboxamide skeleton. The mycelial growth inhibition experiment showed that compound **M15** exhibited a very good control effect against four plant pathogens, with inhibition rates of more than 60% at a dose of 50 μg/mL. A structure–activity relationship study found that N-O-benzyl-substituted derivatives showed better antifungal activity than others, especially the introduction of a halogen on the benzyl. Furthermore, the molecular docking results suggested that π–π interactions with Trp35 and hydrogen bonds with Tyr33 and Trp173 were crucial interaction sites when inhibitors bound to SDH. Morphological observation of mycelium revealed that **M15** could inhibit the growth of mycelia. Moreover, in vivo and in vitro tests showed that **M15** not only inhibited the enzyme activity of SDH but also effectively protected rice from damage due to *R. solani* infection, with a result close to that of the control at a concentration of 200 μg/mL. Thus, the *N*-(alkoxy)diphenyl ether carboxamide skeleton is a new starting point for the discovery of new SDH inhibitors and is worthy of further investigation.

## 1. Introduction

Currently, the top three challenges for companies focused on R&D are resistance and its impact on the performance of agrochemicals, increasing regulatory safety margins, and product costs [1,2]. In addition, there are a large number of patents that claim various compounds with pesticide activity or pharmaceutical activity. Therefore, it is increasingly difficult to obtain a novel and structurally simple molecular skeleton with pesticide activity. Targeted pesticide molecular design can help us overcome the current challenges and invent selective, environmentally benign, low-use-rate, and cost-effective active ingredients [2,3,4].

**Figure 1:**
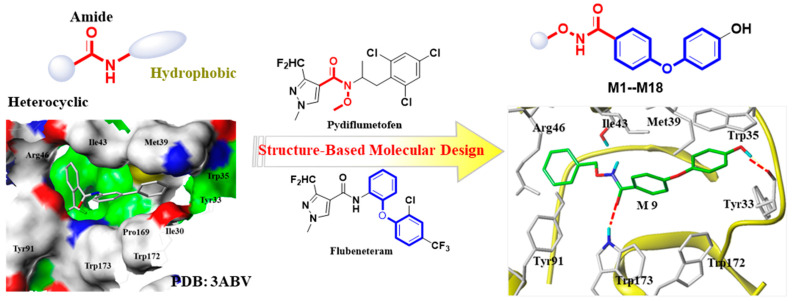
The pharmacophore of targeted SDH inhibitors and the design strategy of *N*-(alkoxy)diphenyl ether carboxamide derivatives.

Succinate dehydrogenase (SDH, EC 1.3.5.1), also known as succinate ubiquinone oxidoreductase (SQR), is a very important target for fungicide development. The biological function of SDH is very clear. Simply put, SDH is located in the inner mitochondrial membrane and can transfer an electron from succinate to fumarate, accompanied by the reduction of ubiquinone to ubiquinol [5,6]. To date, the crystal structure of the SDH protein from different species and the complex structure of the protein with an inhibitor have been obtained [7,8,9]. The structure of SDH is composed of four subunits: hydrophilic subunits flavoprotein (FP, SDHA), iron–sulfur protein (Ip, SDHB), and two membrane anchor proteins (CybL, SDHC; CybS, SDHD). In general, the inhibitors act in the SDHB subunit and block electron transfer, resulting in the death of the pathogen [10]. There are more than 20 commercial succinate dehydrogenase inhibitor (SDHI) fungicides. A common feature is that amide fragments are present in all commercialized SDH inhibitors (Figure 1). The heterocyclic fragment is connected to the carbonyl group, and the N atom at the other end is usually connected to a hydrophobic group [11]. Pydiflumetofen, an efficient and broad-spectrum SDHI fungicide, was marketed by Syngenta in 2017 and has been registered for use in potatoes, soybean, oilseed rape, grapes, and peanuts [12]. However, the risk of Pydiflumetofen resistance develops quickly in *Fusarium asiaticum* [13]. Most of the targeted SDH agents, such as thifluzamide, have also led to the resistance of pathogens during use, which also encourages us to develop novel SDH fungicides [14].

The heterocyclic amide skeleton is a well-known core pharmacophore fragment of targeted succinate dehydrogenase (SDH) inhibitors. Consequently, many researchers have primarily focused on modifying the heterocyclic acid core and hydrophobic side moiety (Figure 1). However, our research has been centered on identifying novel pharmacophore structures that could replace amide-bond linkers. Recently, pydiflumetofen, a fungicide developed and marketed by Syngenta, exhibited broad-spectrum and efficient fungicidal activity. Notably, the N-methoxy group in this fungicide is the first application in the targeted SDH fungicide, which has piqued our interest. In addition, as a popular functional scaffold, diaryl ethers are widely used in medicine and pesticide research [15,16]. For example, flubeneteram, a novel candidate fungicide, exhibited a broad-spectrum antifungal activity due to the introduction of a diphenyl ether fragment [5,17].

Upon analyzing the cocrystal structures of the SDH–flutolanil derivative (PDB: 3ABV, Figure 1), we observed that the active pocket of SDH is a long and narrow barrel structure. Hydrogen bond interactions and hydrophobic interactions are two key interactions when inhibitors bind to SDH [18]. Therefore, we used a structure-based molecular design strategy to design a series of derivatives containing an *N*-(alkoxy)diphenyl ether carboxamide skeleton. As hypothesized, the carboxamide fragment could form hydrogen bonds with Trp173, and the terminal benzene ring formed π–π interactions with Trp35 in the molecular docking (Figure 1). In this study, we synthesized target compounds **M1**–**M18**, evaluated their antifungal activity against four types of phytopathogenic fungi, and investigated the mechanism of these compounds through morphological analysis, molecular docking, and SDH inhibitory activity evaluation.

## 2. Results and Discussion

### 2.1. Molecular Design and Synthesis

**Figure 2:**
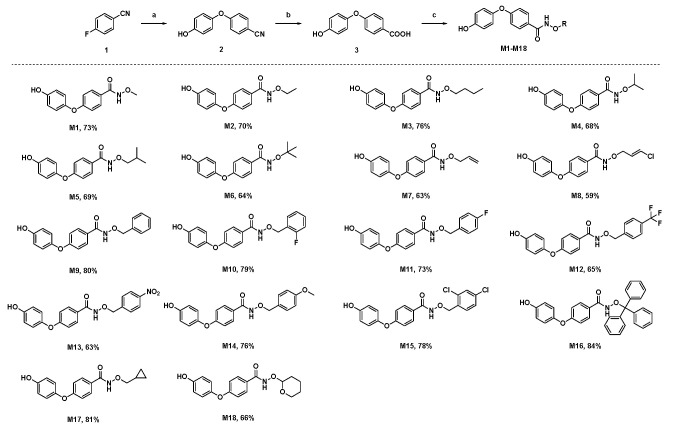
The chemical structure and synthesis scheme of target compounds **M1**–**M18**. Reagents and conditions: (a) hydroquinone, K_2_CO_3_, DMF, 100 °C; (b) NaOH, THF: H_2_O (1:1), reflux; (c) amino derivative, HATU, Et_3_N. R stands for an alkyl, (un)substituted benzyl, heterocyclic ring.

Here, we added a structure-based molecular design strategy to design a series of derivatives containing an *N*-(alkoxy)diphenyl ether carboxamide skeleton (**M1**–**M18**, Figure 1). Meanwhile, a molecular docking study showed that compound **M9** can be well-bound in the active pocket of porcine SDH, and the binding model is similar to that of the SDH–flutolanil derivative (PDB: 3ABV, Figure 1). As expected, the terminal OH group interacts with Tyr33 to form a hydrogen bond. The diphenyl ether fragments form strong hydrophobic interactions with Trp35, Trp172, and Trp173. In addition, benzyl groups can also form hydrophobic interactions with neighboring amino acids. The result of the molecular docking suggested that the *N*-(alkoxy)diphenyl ether carboxamide skeleton has the potential to become a new class of targeted SDH inhibitors. Thus, we tried to design a convenient and efficient synthesis scheme and finally obtain the target compound. The general synthesis procedure of compounds **M1**–**M18** is shown in Figure 2. 4-Fluorobenzonitrile was used as the starting material and reacted with hydroquinone to obtain a diphenyl ether skeleton. Under alkaline conditions, CN was hydrolyzed to a carboxyl group, which further reacts with substituted N-O derivatives to synthesize the target compound [19]. During the experiment, we found that the conversion rate of the reaction was high, but the yield of the target compound was not ideal. During the silica gel column chromatography process (eluents: petroleum ether and ethyl acetate), probably due to the hydrogen bond interactions between molecules, we found that a small amount of unreacted alkoxy amine intermediate would mix with our target compound, and we had to discard a part of the target compound with lower purity, resulting in a generally low separation yield of our final compound. The chemical structures of all of the synthesized title compounds were confirmed by ^1^H NMR and ^13^C NMR spectroscopy and HRMS (Supplementary Materials).

### 2.2. Antifungal Activity and Structure–Activity Relationship

**Figure 3:**
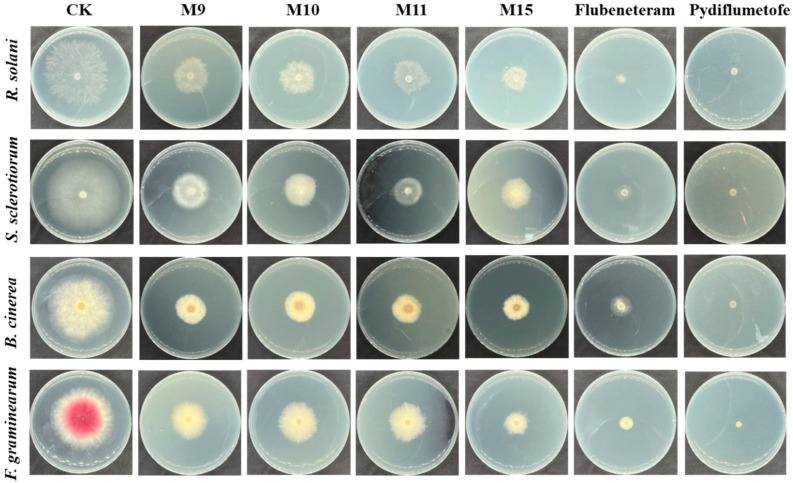
The antifungal activity of compounds **M5**, **M9**, **M11**, **M15**, flubeneteram and pydiflumetofe in a petri dish at a dose of 50 μg/mL. A 0.5% DMSO aqueous solution was used as a blank control group.

Adopting the mycelial growth inhibition method, we evaluated the antifungal activity of all the target compounds against four major crop disease pathogens [20]. We chose a high-efficiency and broad-spectrum fungicide variety, flubeneteram and pydiflumetofen, as the positive control and 0.5% DMSO aqueous solution as the blank treatment group [5]. Compounds **M5**, **M9**, **M11**, and **M15** had obvious inhibitory effects on the mycelial growth of the tested pathogens, but the inhibitory effect was lower than that of the control agents (flubeneteram and pydiflumetofe) at a dose of 50 μg/mL (Figure 3). The cross method was used for statistical analysis, and compound **M15** showed the best antifungal activity against the four kinds of pathogenic bacteria, remaining above 60% (Table 1). The other compounds, **M9**, **M10**, and **M11**, were able to achieve antibacterial activity of more than 50% against three of these pathogens.

Furthermore, we found that the R group had a significant effect on fungicidal activity (Table 1). For the alkyl-substituted derivatives **M1**–**M6**, compound **M5** (R = isobutyl) showed 47% antifungal activity against *B. cinerea,* which is better than other alkyl-substituted compounds. With an increasing substituent chain length, the antifungal activity also increased slightly (butyl > methyl), but the increase in the spatial volume of the alkyl substituents was not conducive to the improvement in antifungal activity (isobutyl > tert-butyl). Introducing allyl (**M7**) and 3-chloroallyl (**M8**) did not significantly improve the antifungal activity of this compound against the four types of pathogenic fungi compared to the alkyl-substituted compounds. Surprisingly, for the benzyl-substituted derivatives **M9**–**M16**, most of the compounds showed good antifungal activity. In particular, the mycelial growth inhibition rate of **M15** (2,4-dichlorobenzyl) was more than 70% for *R. solani*. We found that adding halogen to the benzyl group was beneficial for increasing antifungal activity. For example, compounds **M10** (2-fluorobenzyl), **M11** (4-fluorobenzyl), and **M15** showed better antifungal activity than **M9** (benzyl) against *S. sclerotiorum*, *B. cinerea*, and *F. graminearum*. In addition, introducing electron-withdrawing groups (-CF_3_ and -NO_2_) and electron-donating groups (-OCH_3_), the antifungal activity of these compounds is lower than that of compound **M9**. Furthermore, for compounds **M16** (tri-phenylmethyl), **M17** (cyclopropylmethyl), and **M18** (tetrahydro-2H-pyran-2-yl), the inhibitory effect on the mycelium of the four tested pathogens was lower than that of benzyl substituted compound **M9**.

The results of the structure–activity relationship study suggested that the benzyl scaffold played an integral role in enhancing the antifungal activity of the compounds. In particular, introducing one or two halogen atoms on the benzyl ring could further improve the inhibitory potency. It is worth mentioning that large steric hindrance groups cannot increase the antifungal activity of the compounds.

### 2.3. Molecular Docking Models of the Flutolanil Derivative, M1, M9, and M15 with Porcine SDH (PDB ID 3ABV)

To further clarify the reasons for the difference in antifungal activity between alkyl-substituted and benzyl-substituted compounds, we studied the differences in the binding mode and binding stability of the compounds to the target via molecular docking. Currently, the crystal structure of SDH is mainly derived from animals and microorganisms. Although the homology of SDH from different species varies greatly, the key amino acid sites in the catalytic active cavity are conserved [7,8,21]. Thus, the porcine SQR protein is used as the receptor, and the porcine SDH–flutolanil derivative complex (PDB: 3abv) serves as a reference and is used to define the binding pocket of the inhibitor [15,18]. The molecular docking process was carried out in SYBYL-X 2.0 by running surflex-dock.

**Figure 4:**
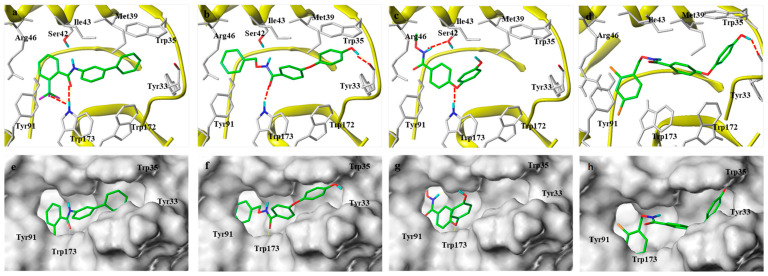
Molecular docking models of the flutolanil derivative (**a**), **M9** (**b**), **M1** (**c**), and **M15** (**d**) with porcine SDH (PDB ID 3ABV) and surface models are shown in (**e**–**h**), respectively. Green represents the inhibitor molecule, and white represents the amino acid residues. The red dotted lines indicate hydrogen bonding interactions.

In the co-crystal structure, the binding model of SDH–flutolanil derivative is taken as reference, and amino acid residues within a distance of 10Å around the ligand are defined as binding pockets. The molecular docking results are shown in Figure 4; in the binding mode of the flutolanil derivative and **M9**, both the hydrogen bond interaction with Trp173 and the hydrophobic interaction with Trp35 are maintained. We also observed the halogen bond between -CF_3_ and Trp173 in the binding mode of the flutolanil derivative, which may be the reason why the introduction of F atoms can increase the antifungal activity of **M11** or **M10**. The hydrogen bonding interactions between -OH and TYR33 in the M9 model can further increase bond stability, and the binding energy of SDH-**M9** (7.80 kcal/mol) is better than that of the flutolanil derivative–SDH (7.13 kcal/mol). There is a slight difference in the binding model of SDH-**M15** (Figure 4d, 7.03 kcal/mol). In the active pocket of SDH, although the hydrogen bond between OH and Trp35 is preserved, the hydrogen bond between the carbonyl group and Trp173 is lost. However, in the binding mode of SDH-**M1**, the oxygen atom on the diphenyl ether, rather than the carbonyl oxygen atom, forms a hydrogen bond with Trp173. In addition, the hydrogen bonding between -OH and TYR33 is lost, and NH forms a hydrogen bond with Ser42 instead (Figure 4c). The loss of π–π interactions with Trp35 and hydrogen bonds with Tyr33 directly leads to a significant reduction in the binding stability of the molecule and SDH, and the binding energy of SDH-**M1** is only 4.54 kcal/mol. Furthermore, comparing the surface models of **M1**, **M9**, or **M15** with SDH**, M1** occupies only 1/2 of the space in the active pocket of SDH, resulting in a lower binding stability with the target than **M9** or **M15**. Thus, the introduction of benzyl can improve the bind stably in the active pocket of SDH, which may be one of the reasons why the antifungal activity of **M1** is lower than that of **M9** or **M15**.

### 2.4. SEM Analysis

**Figure 5:**
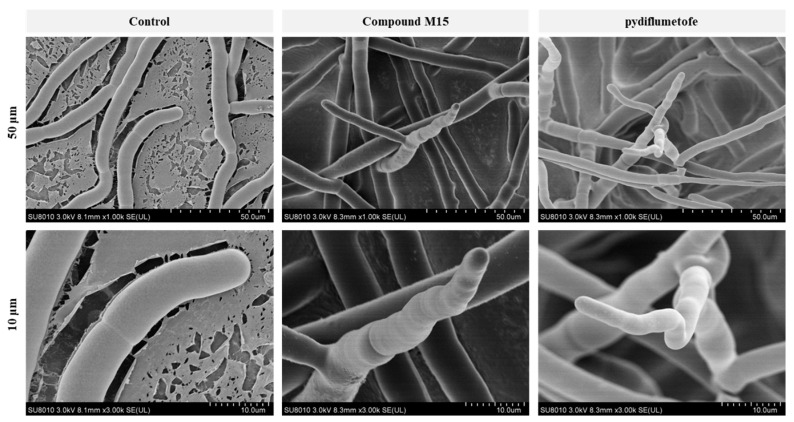
SEM of the hyphal surfaces of *R. solani* in the blank control (DMSO), **M15**, and pydiflumetofe at a dose of 50 μg/mL. The resolution ratios of the graphs were 50 μm and 10 μm.

We selected **M15** with the best antifungal activity to observe its effect on the mycelial growth and morphology of *R. solani*. As shown in Figure 5, after treatment with **M15** and pydiflumetofe at a dose of 50 μg/mL, the growth morphology of mycelium showed some obvious common characteristics, such as crumpling, collapse, branching, and hyphal apex growth inhibition. However, for the blank control, the hyphae morphology of *R. solani* grew vigorously and stretched well, and the surface of the hyphae was relatively smooth and regular. We did not observe hyphal branching. Thus, **M15** and pydiflumetofe can affect the cell wall and membrane of hyphae, resulting in mycelial growth inhibition [22,23].

### 2.5. In Vitro SDH Inhibitory Activity Assay

**Figure 6:**
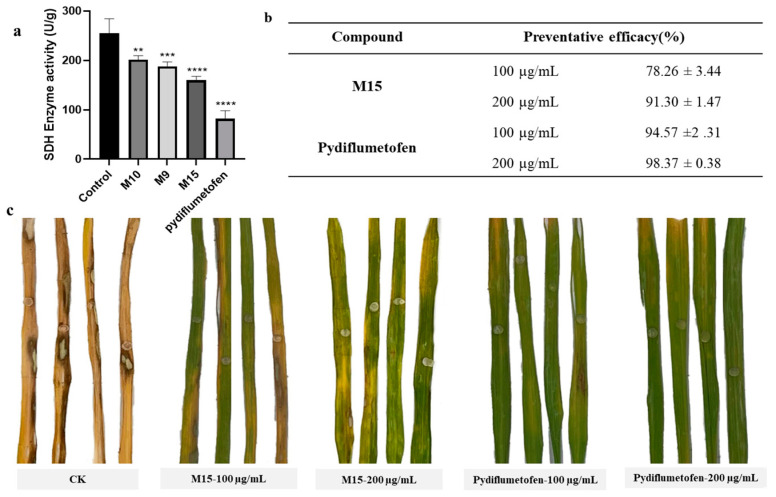
The activity assay of **M15** and pydiflumetofen against *R. solani* in vivo and in vitro. (**a**) In vitro SDH inhibitory activity assay. One-way analysis of variance was used, and all data were compared with the control, ** *p* =0.0052, *** *p* =0.0009, **** *p* ≤ 0.0001. (**b**,**c**) The protective effect of **M15** and pydiflumetofen on rice sheath blight.

According to the results of the mycelial growth inhibition rate experiment, compounds with an inhibition rate greater than 50% were selected to test their inhibitory activity on the SDH enzyme. We further tested the inhibitory activity of **M9**, **M10**, and **M15** on *R. solani* SDH by utilizing the succinate dehydrogenase (SDH) activity assay kit [24,25]. The dose of agent used was 50 μg/mL. Pydiflumetofe was used as the positive control. As shown in Figure 6a, all compounds could inhibit the activity of SDH in *R. solani* mycelia. And the inhibitory effect of enzyme activity was consistent with that of the compound on hyphal growth (**M15** > **M9** >**M10**). At the same dose, the inhibition potency of **M15** was not better than that of the commercial agent. Combined with the results of molecular docking and morphological observation experiments of mycelia, we confirmed that the target of this class of compounds is SDH.

### 2.6. In Vivo Antifungal Activity

Finally, the antifungal activity of **M15** against *R. solani* was tested on the leaves of rice (Figure 6b,c). After 5 days of inoculation, the disease spots on the leaves of the blank treatment group were larger, and the leaves were completely withered. Under the same culture conditions, the leaves treated with the agent had fewer spots, and the leaves were still green. Statistical analysis of the lesion area showed that the protective effect of **M15** (91.30%) was close to that of the positive control agent (98.37%) at a treatment concentration of 200 μg/mL. As the concentration of the agent used decreased, the protective effect of **M15** also decreased, with only 78.26% antifungal activity at a dose of 100 μg/mL. Thus, **M15** can be regarded as a leader structure for a novel class of targeted SDH inhibitors.

## 3. Materials and Methods

### 3.1. Chemical Reagents and Instruments

The reagents and alkoxy amine intermediates used in chemical synthesis were purchased directly from the company, such as Macklin, Aladdin, Energy chemical, etc. All solvents were of chemical purity and without further processing, such as petroleum ether (PE), ethyl acetate (EA), dimethylformamide (DMF), dichloromethane (DCM), tetrahydrofuran (THF). Usually, the reactions were monitored by thin-layer chromatography (TLC, Qingdao Marine Chemical Inc., Qingdao, China). The structure of the compounds was confirmed with ^1^H NMR, ^13^C NMR spectra, and high-resolution mass spectra (HRMS, Agilent 6210 TOF LC-MS spectrometer, Agilent, Santa Clara, CA, USA). ^1^H NMR and ^13^C NMR spectra were collected with JNM-ECZ500R (500 MHz, Jeol, Tokyo, Japan) with CDCl_3_ or DMSO-*d*_6_ as solvents and tetramethylsilane (TMS) as internal standard; chemical shift values (δ) were listed in parts per million (ppm). The spectra were analyzed with MestReNova 14.0.

### 3.2. General Synthesis Method of Intermediate 2

Hydroquinone (1.65 g, 15 mmol) and potassium carbonate (2.76 g, 20 mmol) were added into 20 mL solvent DMF, stirred and heated to approximately 110℃ for 30 min; then raw material 4-fluorobenzonitrile (1.21 g, 10 mmol) was added, continuing the reaction for 4 h, and it was monitored via TLC. After the reaction completion, 80 mL water was added to the reaction system and then extracted with ethyl acetate three times. Then, the organic phase was washed in saturated salt water. Finally, solid intermediate 2 was obtained after drying with anhydrous sodium sulfate and spin-drying the solvent.

### 3.3. General Synthesis Method of Intermediate 3

Intermediate 2 (1.05 g, 5 mmol) and sodium hydroxide (0.80 g, 20 mmol) were dissolved in 20 mL THF:H_2_O (1:1), stirred and refluxed for 12 h. TLC was used to monitor the end of the reaction process. Then, the solution was returned to room temperature after reaction completion, the pressure was reduced, and the THF was removed. The remaining reaction liquid was adjusted to pH = 1–2 with 2 N dilute hydrochloric acid. Intermediate 3 was obtained after filtration.

### 3.4. General Synthesis Method of Title Compounds ***M1***–***M18***

Intermediate 3 (1 mmol) and N-alkoxy intermediates (2 mmol) were dissolved in DCM (5 mL), and then O-(7-azabentriazole-1-acyl)-N,N,N′,N′-tetramethylammonium hexafluorophosphate (HATU) (1.1 mmol) and triethylamine (2 mmol) were added. Then, the solution was stirred at room temperature for 3 h, and TLC monitoring was employed. At the end of the reaction, saturated NaHCO_3_ solution was added and extracted three times with DCM. The organic layer was dried with anhydrous sodium sulfate and concentrated under reduced pressure. The crude product was further purified via column chromatography (petroleum ether/ethyl acetate = 3/1). Structural characterization data of target compounds are shown in the Supplementary Materials.

### 3.5. Antifungal Activity Screening

The effects of the target compounds on *Rhizoctonia solani*, *Sclerotinia sclerotiorum*, *Fusarium graminearum*, and *Botrytis cinerea* were determined using the mycelial growth rate method. The compounds were dissolved in DMSO and added to quantitative PDA (20 g glucose, 200 g potato, 20 g agar, and 1 L ultrapure water) medium to prepare a drug-containing plate with a final concentration of 50 μg/mL. DMSO solvent and water were used as solvent controls and blank controls, respectively. Flubeneteram and pydiflumetofe were used as positive controls. The pathogen-containing cake was inoculated in the centers of the agent-containing plate, blank control plate, and solvent control plate. The samples were cultured in a dark incubator at 25 °C for 3–5 days, and the colony diameter of the samples was calculated by the cross method. All experiments were independently repeated three times. The inhibition rate (%) was calculated as [(control colony diameter − treatment colony diameter)/(control colony diameter − 0.5)] × 100 (0.5 was the diameter of the cake (cm)).

### 3.6. In Vitro SDH Inhibitory Activity Assay

Compounds **M9**, **M10**, and **M15** were tested for their inhibitory activity against SDH by following the operation manual of a succinate dehydrogenase assay kit (BC0955, Beijing Sun Biotechnology Co., Ltd., Beijing, China). The *R. solani* were inoculated in PDB (20 g sugar, 200 g potato, 1 L ultrapure water) medium at 135 rpm and 25 °C for 24 h, then M15 was added, the final concentration was maintained at 50 μg/mL, and the culture was continued for 24 h. Mycelia (0.1 g) were placed in liquid nitrogen and rapidly ground. Then, reagent 1 (1 mL) and reagent 2 (10 μL) were added and mixed well and centrifuged at 11,000× *g* and 4 °C for 10 min. The supernatant was collected and stored at 0 °C. At the same time, reagent 3 (170 μL) and reagent 4 (10 μL) were mixed and incubated at 25 °C for 10 min, and then reagent 5 (10 μL) and sample solution (10 μL) or 10 μL water were added as blank controls. The absorbance of the mixture was monitored in a 96-well plate at 600 nm. The inhibitory effect (U/g) was calculated according to the sample quality. The experiment was repeated three times.

### 3.7. Antifungal Activity In Vivo

Compound **M15** dissolved in 2% DMSO was mixed with 0.03% Tween-80 and prepared as 100 µg/mL and 200 µg/mL solutions. DMSO (2%) and 0.03% Tween-80 aqueous solution were used as blank controls. The commercial fungicide pydiflumetofen was chosen as the positive control. The fresh rice leaves were cut to approximately 15 cm at the tillering stage, and the ligand solution was sprayed on the leaf. After 24 h of treatment, holes were punctured in the middle of the rice leaves with sterile needles, and then *R. solani* fungus cakes with a diameter of 3 mm were inoculated on the holes. The completely inoculated rice leaves were cultivated in a light incubator (25 °C, 90% relative humidity, 12:12 light–dark ratio) for 5 days. The lengths of lesions in each treatment group were measured, and the control effect of each treatment group was calculated. Each treatment was repeated using 15 leaves. The in vivo antifungal activity on rice leaves was calculated as follows:

A(%) = [(C − T)/C] × 100

where A represents the in vivo antifungal activity, C indicates the length of the lesion in the blank control, and T represents the length of the lesion in the chemical treatment.

### 3.8. Molecular Docking

The porcine SQR structure (PDB ID 3ABV) was used as a receptor protein. After completing protein optimization, we defined the binding site of the ligand referring to the binding site of the flutolanil derivative in the cocrystal structure. Referring to SYBYL’s user manual, the other parameters were set to default values. A total of 200 runs were performed using a surflex-dock computing module with the minimum RMSD between final poses ≤ 0.05. The top-ranked conformation was selected as the stable binding conformation.

### 3.9. Scanning Electron Microscopy (SEM) Observations

PDA medium and compound **M15** were mixed and maintained at a final concentration of 50 μg/mL, 0.5% DMSO aqueous solution treatment was used as the blank control, and pydiflumetofe (50 μg/mL) was used as the positive control. *R. solani* was inoculated on the medicated plate and cultured for 48 h. The outermost mycelium blocks were cut from the medium and fixed with glutaraldehyde. Micromorphological observations were carried out through SEM (Hitachi SU8010, Hitachi Co., Tokyo, Japan) at resolution ratios of 100 μm and 10 μm.

## 4. Conclusions

In summary, the prevalence of plant diseases instigated by pathogenic fungi is a significant factor contributing to the reduction in crop yield. Fungicides have been effectively employed to control and minimize these losses. However, the increasing resistance of pathogens, due to the extensive use of fungicides, poses a serious challenge. SDH, a known key target for fungicide development, and the heterocyclic amide skeleton, a core pharmacophore fragment of targeted SDH inhibitors, are of particular interest. In this study, we aimed to identify novel pharmacophore structures that could substitute amide-bond linkers. By integrating the spatial structure characteristics of the SDH active cavity, we utilized a structure-based molecular design strategy to design and synthesize a series of derivatives containing an *N*-alkoxy diphenyl ether carboxamide skeleton (**M1**–**M18**).

The mycelial growth inhibition experiment confirmed that **M15** outperformed other compounds in antifungal activity, inhibiting *R. solani* mycelium growth by over 70%. Additionally, compounds **M9**, **M10**, and **M11** also demonstrated over 50% inhibitory potency against the three tested pathogenic fungi. A structure–activity relationship analysis suggested that the benzyl group is more effective in enhancing antifungal activity than the alkyl or heterocyclic ring (pyran). A comparison of the binding models of SDH-**M9**, SDH-**M15**, and SDH-**M1** revealed that the introduction of benzyl could improve the binding stability in the active pocket of SDH, which may account for the lower antifungal activity of **M1** compared to **M9** or **M15**. SEM observation revealed that **M15** could not only inhibit the growth of *R. solani* hyphae but also affect the growth morphology of the mycelium, similar to the effect of pydiflumetofe treatment. In vitro assays showed that **M15** inhibited the activity of SDH in *R. solani*, further confirming that the compound operates in the same manner as pydiflumetofe, acting on SDH.

Moreover, following treatment with **M15**, the protective effect against *R. solani* reached 91.30% in rice leaves at a dose of 200 μg/mL, closely mirroring the effect of the positive control of pydiflumetofe. These results suggest that the N-alkoxy carboxamide skeleton can function as a novel pharmacophore for targeting-SDH inhibitors to replace existing amide-bond linkers. Both in vitro and in vivo tests indicate that compound **M15** has significant potential as a novel fungicide targeting SDH.

## Figures and Tables

**Table 1:** Mycelial growth inhibition rate by compounds **M1**–**M18**.

No.	R	Inhibition Rate ± Standard Deviation (SD) %, 50 μg/mL			
		*R. solani*	*S. sclerotiorum*	*B. cinerea*	*F. graminearum*
**M1**	methyl	13.09 ± 3.93 n	18.10 ± 1.10 j	32.17 ± 1.98 h	2.92 ± 2.62 l
**M2**	ethyl	3.11 ± 1.67 q	21.63 ± 1.77 i	11.14 ± 0.74 l	11.31 ± 1.20 j
**M3**	butyl	25.78 ± 1.40 j	35.67 ± 2.90 f	37.65 ± 0.99 g	25.91 ± 1.65 fg
**M4**	isopropyl	1.86 ± 0.96 q	16.85 ± 2.75 j	1.20 ± 2.72 o	2.92 ± 3.58 l
**M5**	isobutyl	34.47 ± 1.40 h	36.52 ± 3.64 f	47.89 ± 1.36 f	27.37 ± 1.65 f
**M6**	tert-butyl	2.17 ± 1.02 q	21.91 ± 2.54 i	2.11 ± 1.36 o	14.6 ± 2.77 i
**M7**	allyl	28.88 ± 2.18 i	25.00 ± 6.28 h	4.52 ± 2.11 n	10.58 ± 2.15 j
**M8**	(*E*)-3-chloroallyl	25.47 ± 1.18 j	27.25 ± 2.48 g	11.75 ± 2.11 kl	25.18 ± 2.15 g
**M9**	benzyl	56.83 ± 1.40 d	57.02 ± 1.77 e	62.35 ± 2.11 e	43.80 ± 1.13 e
**M10**	2-fluorobenzyl	52.80 ± 2.81 e	64.04 ± 0.87 d	62.65 ± 0.93 e	43.43 ± 2.56 e
**M11**	4-fluorobenzyl	47.83 ± 1.67 f	69.10 ± 2.04 c	64.46 ± 0.93 d	51.09 ± 2.26 d
**M12**	4-(trifluoromethyl)benzyl	38.82 ± 1.40 g	16.57 ± 3.49 j	1.20 ± 1.48 o	6.93 ± 2.30 k
**M13**	4-nitrobenzyl	14.91 ± 2.26 m	25.56 ± 1.27 gh	18.67 ± 1.14 j	20.80 ± 2.15 h
**M14**	4-methoxybenzyl	18.01 ± 2.04 l	17.13 ± 1.97 j	2.41 ± 1.62 o	2.92 ± 2.26 l
**M15**	2,4-dichlorobenzyl	74.22 ± 1.40 c	63.20 ± 3.09 d	68.07 ± 0.93 c	68.98 ± 0.89 c
**M16**	tri-phenylmethyl	23.60 ± 2.89 k	22.19 ± 3.09 i	8.13 ± 2.11 m	20.44 ± 2.26 h
**M17**	cyclopropylmethyl	6.83 ± 1.67 p	27.25 ± 3.60 g	13.25 ± 3.02 k	19.34 ± 1.65 h
**M18**	tetrahydro-2*H*-pyran-2-yl	8.70 ± 1.67 o	21.63 ± 1.77 i	29.82 ± 1.78 i	14.96 ± 2.15 i
Flubeneteram		91.30 ± 0.96 b	83.99 ± 0.92 b	74.10 ± 1.48 b	85.40 ± 1.79 b
pydiflumetofe		100 a	100 a	100 a	100 a

Data are reported as average of three biological replicates ± SD. Different letters correspond to treatments that differ for Tukey’s HSD test for *p* < 0.05, which indicate significant differences within treatments and references at the same concentration.

## Data Availability

The data presented in this study are available in the article and Supplementary materials.

## Supplementary Materials

The following supporting information can be downloaded at: https://www.mdpi.com/article/10.3390/molecules29010083/s1.
