# Supplementary material for: Design, Synthesis, and Antifungal Activity of N-(alkoxy)-Diphenyl Ether Carboxamide Derivates as Novel Succinate Dehydrogenase Inhibitors
Source: Molecules. 2023 Dec 22;29(1):83. doi: 10.3390/molecules29010083 (PMC10780015; doi:10.3390/molecules29010083)
Supplement: Supplementary file 1 [file molecules-29-00083-s001.zip › molecules-2747140-supplementary.pdf]

Article

Design, Synthesis, and Antifungal Activity of *N*-(alkoxy)-Diphenyl Ether Carboxamide Derivates as Novel Succinate Dehydrogenase Inhibitors

Bo He [1,2], Yanhao Hu [1,2], Wang Chen [1,2], Xu He [1,2], Enpei Zhang [1,2], Mengxu Hu [1,2], Pu Zhang [3], Wei Yan [1,2] and Yonghao Ye [1,2,*]

1 State & Local Joint Engineering Research Center of Green Pesticide Invention and Application, College of Plant Protection, Nanjing Agricultural University, Nanjing 210095, China; hebo@njau.edu.cn (B.H.); huyh@stu.njau.edu.cn (Y.H.); 2022102110@stu.njau.edu.cn (W.C.); 2022102112@stu.njau.edu.cn (X.H.); 12121413@stu.njau.edu.cn (E.Z.); 2021102127@stu.njau.edu.cn (M.H.); yanwei@njau.edu.cn (W.Y.)
2 Key Laboratory of Integrated Management of Crop Diseases and Pests, Ministry of Education, Nanjing 210095, China
3 Research & Development Center, Jiangsu Flag Chemical Industry Co., Ltd., Nanjing 210095, China; zhangpu@flagchem.com
* Correspondence: yeyh@njau.edu.cn

S1. Structural characterization data of target compounds M1-M18

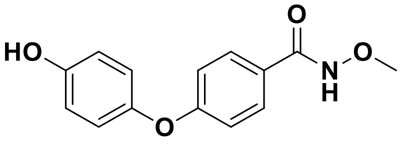

M1

4-(4-hydroxyphenoxy)-N-methoxybenzamide (**M1**); Yield: 73% (189.1 mg); White solid; ^{1}H NMR (500 MHz, DMSO-*d*$_6$) δ 11.66 (s, 1H), 9.48 (s, 1H), 7.73 (d, J = 8.5 Hz, 2H), 6.98 – 6.89 (m, 4H), 6.81 (d, J = 8.0 Hz, 2H), 3.68 (s, 3H).; ^{13}C NMR (125 MHz, DMSO-*d*$_6$) δ 164.09, 161.82, 154.98, 147.28, 129.64, 126.31, 122.12, 116.94, 116.54, 63.78.; ESI-HRMS: m/z [M+H]$^+$ calcd. for [$C_{14}H_{13}NO_4H$]: 260.0917. Found: 260.0918.

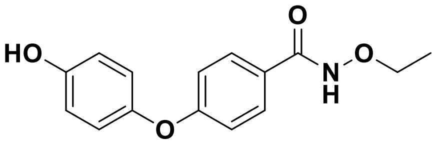

M2

N-ethoxy-4-(4-hydroxyphenoxy)benzamide (**M2**); Yield: 70% (191.2 mg); White solid; ^{1}H NMR (500 MHz, DMSO-*d*$_6$) δ 11.48 (s, 1H), 9.42 (s, 1H), 7.69 – 6.73 (m, 2H), 6.90 – 6.83 (m, 4H), 6.79 – 6.73 (m, 2H), 3.89 – 3.81 (m, 2H), 1.50 (t, J = 3.0, 3H).; ^{13}C NMR (125 MHz, DMSO-*d*$_6$) δ 164.21, 161.75, 154.96, 147.35, 129.66, 126.49, 122.07, 116.94, 116.56, 71.21, 14.07.; ESI-HRMS: m/z [M+Na]$^+$ calcd. for [$C_{15}H_{15}NO_4Na$]: 296.0893. Found: 296.0894.

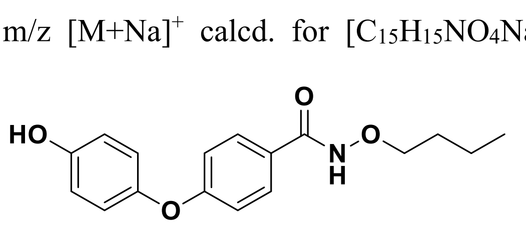

M3

N-butoxy-4-(4-hydroxyphenoxy)benzamide (**M3**); Yield: 76% (228.9 mg); White solid; ^{1}H NMR (500 MHz, DMSO-d_6) δ 11.49 (s, 1H), 9.46 (s, 1H), 7.73 (d, J = 8.5 Hz, 2H), 6.92 (t, J = 9.0 Hz, 4H), 6.81 (d, J = 8.5 Hz, 2H), 3.86 (t, J = 6.5 Hz, 2H), 1.61-1.53 (m, 2H), 1.45-1.35 (m, 2H), 0.91 (t, J = 7.0 Hz, 3H).; ^{13}C NMR (125 MHz, DMSO-d_6) δ 164.20, 161.74, 154.96, 147.35, 129. 65, 126.52, 122.06, 116.93, 116.55, 75.43, 30.30, 19.22, 14.28.; ESI-HRMS: m/z $[M+Na]^+$ calcd. for $[C_{17}H_{19}NO_4Na]$: 324.1206. Found: 324.1207.

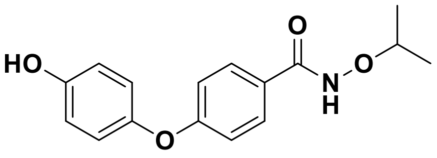

M4

4-(4-hydroxyphenoxy)-N-isopropoxybenzamide (**M4**); Yield: 68% (195.2 mg); W hite solid; ^{1}H NMR (500 MHz, DMSO-d_6) δ 11.35 (s, 1H), 9.43 (s, 1H), 7.74 (d, J = 9.0 Hz, 2H), 6.97 – 6.88 (m, 4H), 6.81 (d, J = 9.0 Hz, 2H), 4.14-4.0 6 (m, 1H), 1.19 (d, J = 6.5 Hz, 6H).; ^{13}C NMR (125 MHz, DMSO-d_6) δ 16 4.45, 161.67, 154.94, 147.39, 129.70, 126.69, 122.04, 116.93, 116.56, 76.98, 21. 20.; ESI-HRMS: m/z $[M+Na]^+$ calcd. for $[C_{16}H_{17}NO_4Na]$: 310.1050. Found: 31 0.1051.

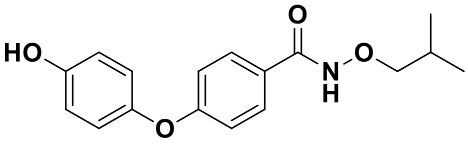

M5

4-(4-hydroxyphenoxy)-N-isobutoxybenzamide (**M5**); Yield: 69% (207.8 mg); Wh ite solid; ^{1}H NMR (500 MHz, DMSO-d_6) δ 11.49 (s, 1H), 9.41 (s, 1H), 7.71 – 7.65 (m, 2H), 6.93 – 6.85 (m, 4H), 6.81 – 6.74 (m, 2H), 3.60 (dd, J = 6.5,

4.0 Hz, 2H), 1.91 – 1.85 (m, 1H), 0.89 (dd, J = 6.5, 4.0 Hz, 6H).; ^{13}C NMR (125 MHz, DMSO-d_6) δ 164.16, 161.73, 154.95, 147.35, 129.65, 126.53, 122.06, 116.93, 116.55, 82.22, 27.40, 19.67.; ESI-HRMS: m/z $[M+Na]^+$ calcd. for $[C_{17}H_{19}NO_4Na]$: 324.1206. Found: 324.1206.

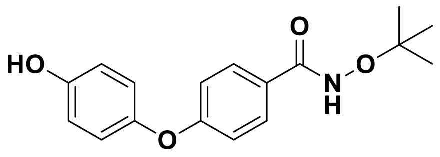

M6

N-(tert-butoxy)-4-(4-hydroxyphenoxy)benzamide (**M6**); Yield: 64% (192.7 mg); White solid; ^{1}H NMR (500 MHz, DMSO-d_6) δ 10.83 (s, 1H), 9.43 (s, 1H), 7.75 (d, J = 9.0 Hz, 2H), 6.92 (dd, J = 8.5, 6.0 Hz, 4H), 6.81 (d, J = 9.0 Hz, 2H), 1.22 (s, 9H).; ^{13}C NMR (125 MHz, DMSO-d_6) δ 166.02, 161.61, 154.90, 147.46, 129.86, 127.03, 121.98, 116.92, 116.56, 81.33, 27.03.; ESI-HRMS: m/z $[M+Na]^+$ calcd. for $[C_{17}H_{19}NO_4Na]$: 324.1206. Found: 324.1205.

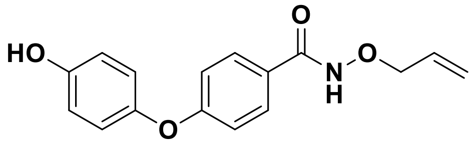

M7

N-(allyloxy)-4-(4-hydroxyphenoxy)benzamide (**M7**); Yield: 63% (179.6 mg); White solid; ^{1}H NMR (500 MHz, DMSO-d_6) δ 11.58 (s, 1H), 9.46 (s, 1H), 7.72 (d, J = 8.5 Hz, 2H), 6.97 – 6.89 (m, 4H), 6.83 – 6.79 (m, 2H), 6.03 – 5.94 (m, 1H), 5.37 – 5.23 (m, 2H), 4.39 (d, J = 6.0 Hz, 2H).; ^{13}C NMR (125 MHz, DMSO-d_6) δ 164.33, 161.79, 154.96, 147.33, 133.75, 129.66, 126.38, 122.07,

119.74, 116.93, 116.57, 76.55.; ESI-HRMS: m/z $[M+Na]^+$ calcd. for $[C_{16}H_{15}NO_4Na]$: 308.0893. Found: 308.0895.

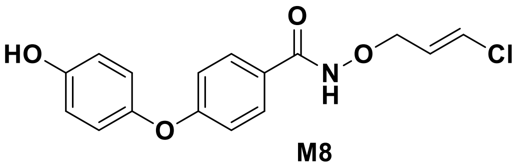

M8

(E)-N-((3-chloroallyl)oxy)-4-(4-hydroxyphenoxy)benzamide (**M8**); Yield: 59% (188.2 mg); White solid; ^{1}H NMR (500 MHz, DMSO-d_6) δ 11.62 (s, 1H), 9.46 (s, 1H), 7.72 (d, J = 8.5 Hz, 2H), 6.93 (dd, J = 9.0, 7.5 Hz, 4H), 6.81 (d, J = 9.0 Hz, 2H), 6.64 (d, J = 13.5 Hz, 1H), 6.16 (dt, J = 13.5, 7.0 Hz, 1H), 4.40 (dd, J = 7.0, 1.5 Hz, 2H).; ^{13}C NMR (125 MHz, DMSO-d_6) δ 164.49, 161.87, 154.97, 147.31, 129.72, 129.49, 126.23, 123.56, 122.08, 116.94, 116.58, 73.46.; ESI-HRMS: m/z $[M+H]^+$ calcd. for $[C_{16}H_{14}ClNO_4H]$: 320.0684. Found: 320.0681.

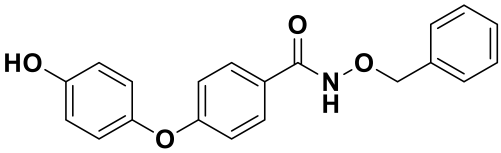

M9

N-(benzyloxy)-4-(4-hydroxyphenoxy)benzamide (**M9**); Yield: 80% (268.1 mg); Yellow solid; ^{1}H NMR (500 MHz, DMSO-d_6) δ 11.69 (s, 1H), 9.47 (s, 1H), 7.74 (dd, J = 8.5, 3.5 Hz, 2H), 7.50 – 7.35 (m, 5H), 6.96 – 6.91 (m, 4H), 6.84 – 6.81 (m, 2H), 4.91 (s, 2H).; ^{13}C NMR (125 MHz, DMSO-d_6) δ 164.48, 161.84, 154.98, 147.34, 136.51, 129.72, 129.45, 128.86, 128.83, 126.40, 122.09, 116.95, 116.58, 77.53.; ESI-HRMS: m/z $[M+Na]^+$ calcd. for $[C_{20}H_{17}NO_4Na]$: 358.1050. Found: 358.1053.

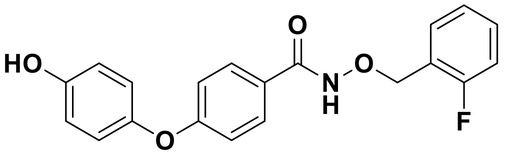

M10

N-((2-fluorobenzyl)oxy)-4-(4-hydroxyphenoxy)benzamide (**M10**); Yield: 79% (27 8.9 mg); White solid; ^{1}H NMR (500 MHz, DMSO-d_6) δ 11.67 (s, 1H), 9.46 (s, 1H), 7.71 (d, J = 8.5 Hz, 2H), 7.52 (t, J = 7.0 Hz, 1H), 7.44 (d, J = 7.0 H z, 1H), 7.24 (t, J = 8.0 Hz, 2H), 6.96 – 6.89 (m, 4H), 6.81 (d, J = 8.0 Hz, 2H), 4.97 (s, 2H).; ^{13}C NMR (125 MHz, DMSO-d_6) δ 164.51, 162.39, 161.84, 160.42, 154.98, 147.30, 132.65, 132.62, 131.42, 131.36, 129.71, 126.31, 124.94, 123.45, 123.33, 122.10, 116.94, 116.54, 115.96, 115.79, 70.82.; ESI-HRMS: m /z [M+Na]$^+$ calcd. for [$C_{20}H_{16}FNO_4Na$]: 376.0956. Found: 376.0957.

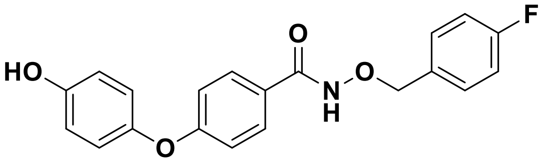

M11

N-((4-fluorobenzyl)oxy)-4-(4-hydroxyphenoxy)benzamide (**M11**); Yield: 73% (25 7.8 mg); White solid; ^{1}H NMR (500 MHz, DMSO-d_6) δ 11.67 (s, 1H), 9.48 (s, 1H), 7.73 (d, J = 8.5 Hz, 2H), 7.50 (dd, J = 8.5, 5.5 Hz, 2H), 7.22 (t, J = 9.0 Hz, 2H), 6.96 – 6.90 (m, 4H), 6.84 – 6.80 (m, 2H), 4.89 (s, 2H).; ^{13}C N MR (125 MHz, DMSO-d_6) δ 164.48, 163.59, 162.85, 161.83, 154.98, 147.32, 1 32.85, 131.75, 131.68, 129.70, 126.33, 122.08, 116.94, 116.57, 115.75, 115.58, 76.65.; ESI-HRMS: m/z [M+Na]$^+$ calcd. for [$C_{20}H_{16}FNO_4Na$]: 376.0956. Found: 376.0957.

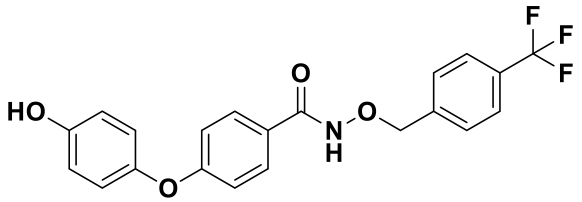

M12

4-(4-hydroxyphenoxy)-N-((4-(trifluoromethyl)benzyl)oxy)benzamide (**M12**); Yield: 65% (262.0 mg); White solid; ^{1}H NMR (500 MHz, DMSO-d_6) δ 11.73 (s, 1H), 9.46 (s, 1H), 7.77 (d, J = 8.0 Hz, 2H), 7.74 – 7.66 (m, 4H), 6.97 – 6.88 (m, 4H), 6.81 (d, J = 7.0 Hz, 2H), 5.01 (s, 2H).; ^{13}C NMR (125 MHz, DMSO-d_6) δ 164.67, 161.90, 154.98, 147.30, 141.42, 129.78, 129.73, 129.30, 129.14, 128.11, 126.18, 125.72, 123.77, 122.08, 116.94, 116.58, 76.56.; ESI-HRMS: m/z $[M+Na]^+$ calcd. for $[C_{21}H_{16}F_3NO_4Na]$: 426.0924. Found: 426.0922.

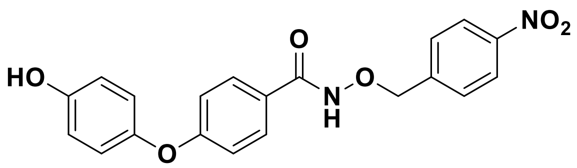

M13

4-(4-hydroxyphenoxy)-N-((4-nitrobenzyl)oxy)benzamide (**M13**); Yield: 63% (239.5 mg); White solid; ^{1}H NMR (500 MHz, DMSO-d_6) δ 11.79 (s, 1H), 9.47 (s, 1H), 8.30 – 8.22 (m, 2H), 7.73 (dd, J = 12.5, 8.5 Hz, 4H), 6.92 (dd, J = 8.5, 6.5 Hz, 4H), 6.81 (d, J = 6.5 Hz, 2H), 5.07 (s, 2H).; ^{13}C NMR (125 MHz, DMSO-d_6) δ 164.69, 161.93, 154.98, 147.79, 147.28, 144.49, 129.99, 129.74, 126.10, 123.96, 122.07, 116.93, 116.59, 76.22.; ESI-HRMS: m/z $[M+Na]^+$ calcd. for $[C_{20}H_{16}N_2O_6Na]$: 403.0901. Found: 403.0902.

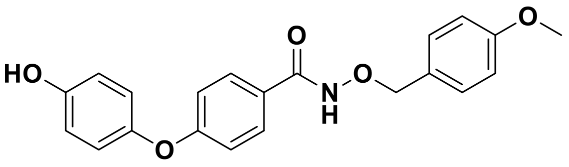

M14

4-(4-hydroxyphenoxy)-N-((4-methoxybenzyl)oxy)benzamide (**M14**); Yield: 76% (277.5 mg); White solid; ^{1}H NMR (500 MHz, DMSO-d_6) δ 11.59 (s, 1H), 9.4 4 (s, 1H), 7.72 (d, J = 9.5 Hz, 2H), 7.36 (d, J = 8.0 Hz, 2H), 6.97 – 6.89 (m, 6H), 6.81 (d, J = 9.0 Hz, 2H), 4.82 (s, 2H), 3.76 (s, 3H).; ^{13}C NMR (12 5 MHz, DMSO-d_6) δ 164.40, 161.79, 159.88, 154.96, 147.33, 131.26, 129.68, 128.43, 126.46, 122.09, 116.93, 116.55, 114.21, 77.16, 55.62.; ESI-HRMS: m/z $[M+Na]^+$ calcd. for [$C_{21}H_{19}NO_5Na$]: 388.1155. Found: 388.1154.

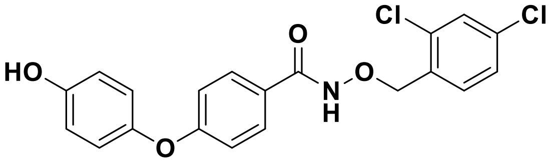

M15

N-((2,4-dichlorobenzyl)oxy)-4-(4-hydroxyphenoxy)benzamide (**M15**); Yield: 78% (314.4 mg); Yellow oily liquid; ^{1}H NMR (500 MHz, DMSO-d_6) δ 11.67 (s, 1 H), 9.45 (s, 1H), 7.70 (d, J = 8.5 Hz, 2H), 7.67 (d, J = 2.0 Hz, 1H), 7.61 (d, J = 8.5 Hz, 1H), 7.48 (dd, J = 8.0, 2.0 Hz, 1H), 6.95 – 6.89 (m, 4H), 6.83 – 6.79 (m, 2H), 5.00 (s, 2H).; ^{13}C NMR (125 MHz, DMSO-d_6) δ 164.67, 161. 89, 154.99, 147.28, 134.81, 134.37, 133.37, 133.26, 129.76, 129.34, 127.97, 12 6.18, 122.10, 116.94, 116.54, 73.60.; ESI-HRMS: m/z $[M+Na]^+$ calcd. for [$C_{20}H_{15}Cl_2NO_4Na$]: 426.0270. Found: 426.0268.

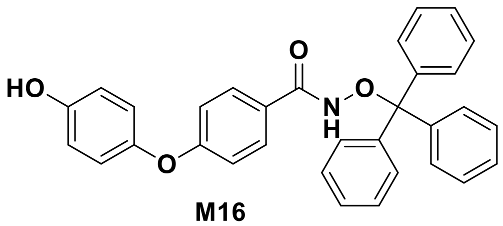

M16

4-(4-hydroxyphenoxy)-N-(trityloxy)benzamide (**M16**); Yield: 84% (409.2 mg); White solid; ^{1}H NMR (500 MHz, DMSO-*d6*) δ 10.77 (s, 1H), 9.44 (s, 1H), 7.46 – 7.24 (m, 17H), 6.96 – 6.73 (m, 6H).; ^{13}C NMR (125 MHz, DMSO-*d6*) δ 166.66, 161.59, 154.90, 147.35, 143.09, 129.98, 129.65, 129.42, 128.02, 126.85, 122.01, 116.91, 116.29, 92.84.; ESI-HRMS: m/z $[M+Na]^+$ calcd. for $[C_{32}H_{25}NO_4Na]$: 510.1676. Found: 510.1677.

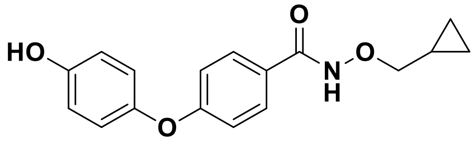

M17

N-(cyclopropylmethoxy)-4-(4-hydroxyphenoxy)benzamide (**M17**); Yield: 81% (242.3 mg); White solid; ^{1}H NMR (500 MHz, DMSO-*d6*) δ 11.60 – 11.49 (m, 1H), 9.49 – 9.40 (m, 1H), 7.75 – 7.68 (m, 2H), 6.97 – 6.86 (m, 4H), 6.84 – 6.76 (m, 2H), 3.72 – 3.63 (m, 2H), 1.08 (s, 1H), 0.57 – 0.45 (m, 2H), 0.25 (dd, J = 12.0, 6.9 Hz, 2H).; ^{13}C NMR (125 MHz, DMSO-*d6*) δ 164.14, 161.72, 154.95, 147.34, 129.63, 126.56, 122.07, 116.93, 116.53, 80.21, 9.79, 3.53.; ESI-HRMS: m/z $[M+Na]^+$ calcd. for $[C_{17}H_{17}NO_4Na]$: 322.1050. Found: 322.1051.

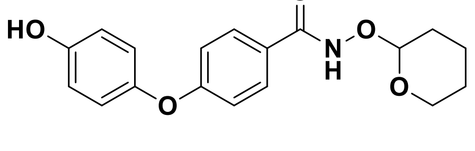

M18

4-(4-hydroxyphenoxy)-N-((tetrahydro-2H-pyran-2-yl)oxy)benzamide (**M18**); Yield: 66% (217.2 mg); White solid; ^{1}H NMR (500 MHz, DMSO-*d₆*) δ 11.55 (s, 1H), 9.46 (s, 1H), 7.75 (d, J = 7.0 Hz, 2H), 6.94 (d, J = 6.4 Hz, 4H), 6.81 (d, J = 6.7 Hz, 2H), 4.97 (s, 1H), 4.05 (s, 1H), 3.51 (d, J = 11.4 Hz, 1H), 1.71 (s, 3H), 1.54 (s, 3H).; ^{13}C NMR (125 MHz, DMSO-d_6) δ 164.30, 161.76, 154.95, 147.37, 129.83, 126.42, 122.04, 116.93, 116.55, 101.52, 61.91, 28.43, 25.26, 1 8.86.; ESI-HRMS: m/z [M+Na]$^+$ calcd. for [$C_{18}H_{19}NO_5Na$]: 352.1155. Found: 3 52.1154.

S2. Spectrums of the representative compounds M1-M18

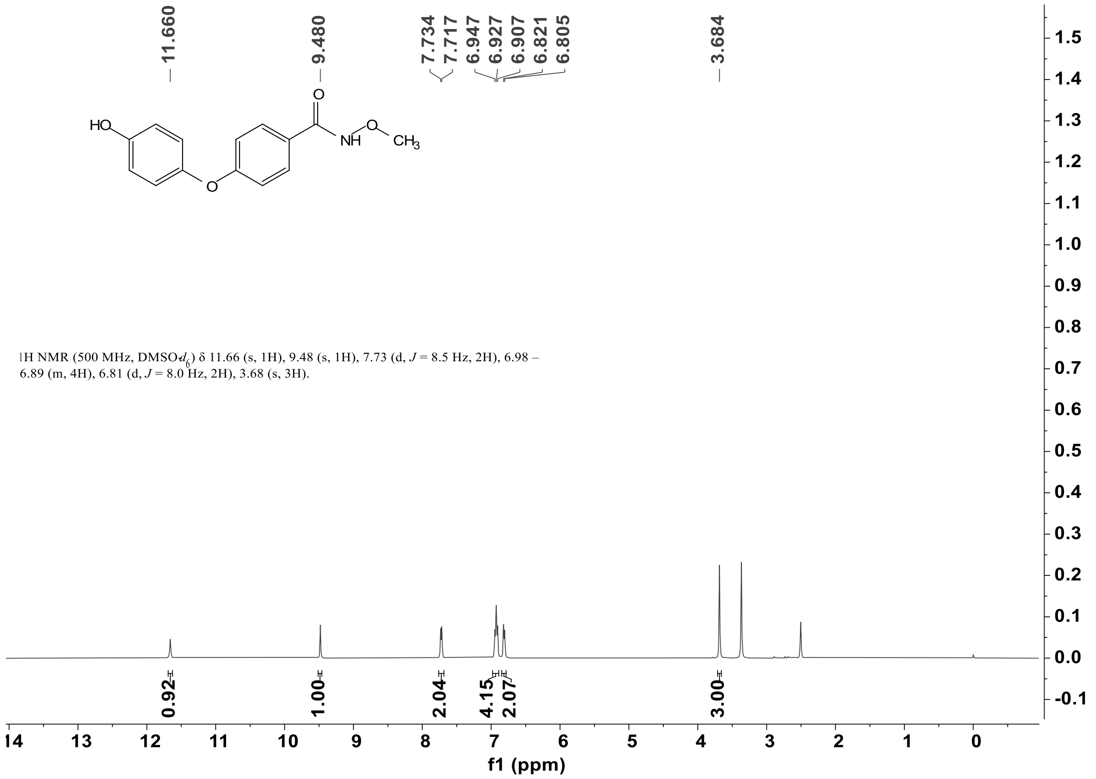

The ^{1}H NMR spectrogram of compound **M1**

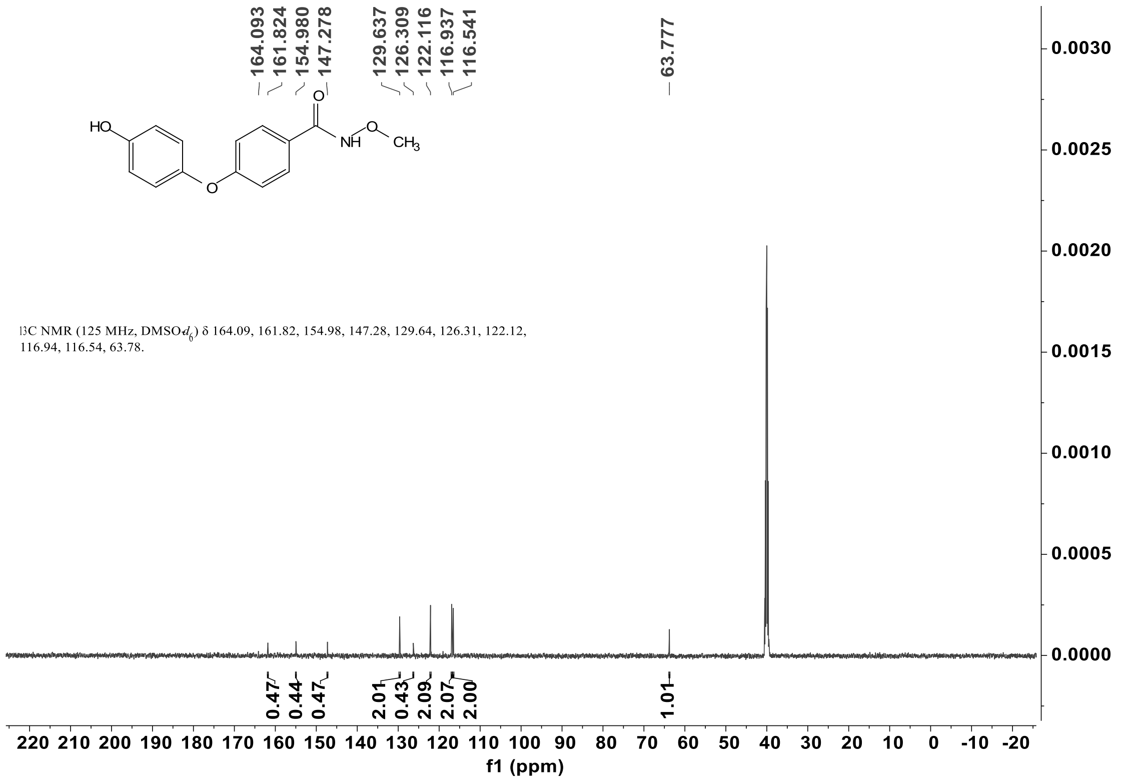

The ^{13}C NMR spectrogram of compound **M1**

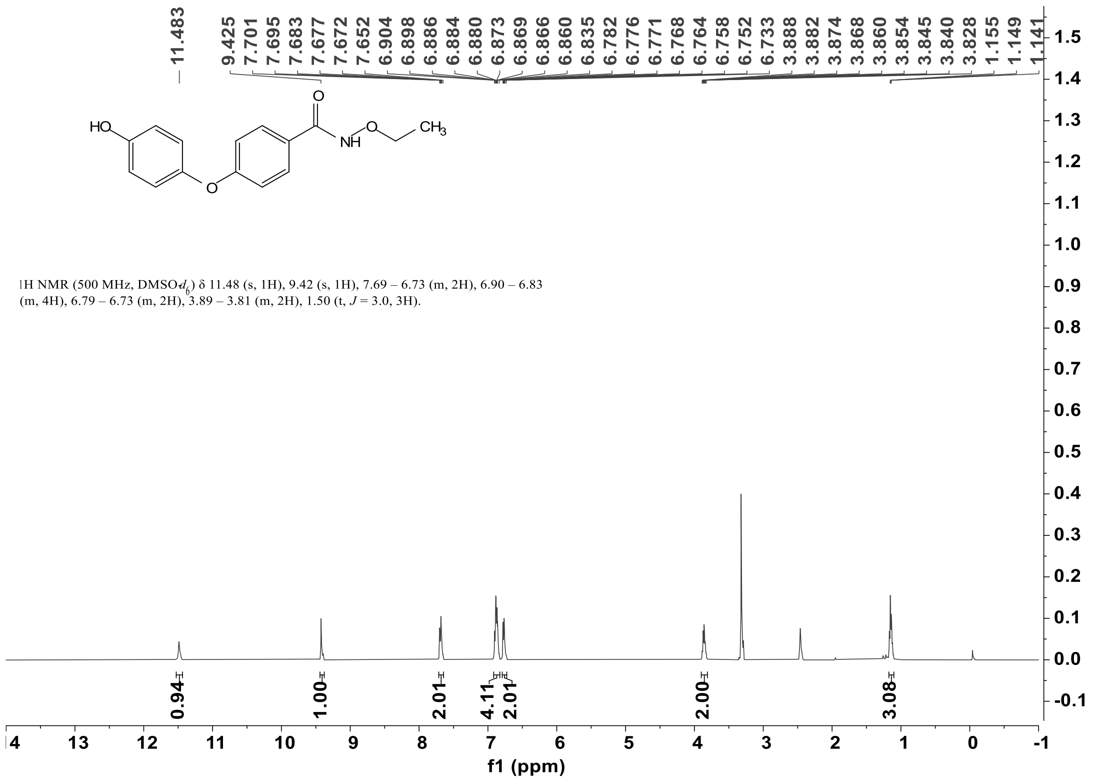

The ^{1}H NMR spectrogram of compound **M2**

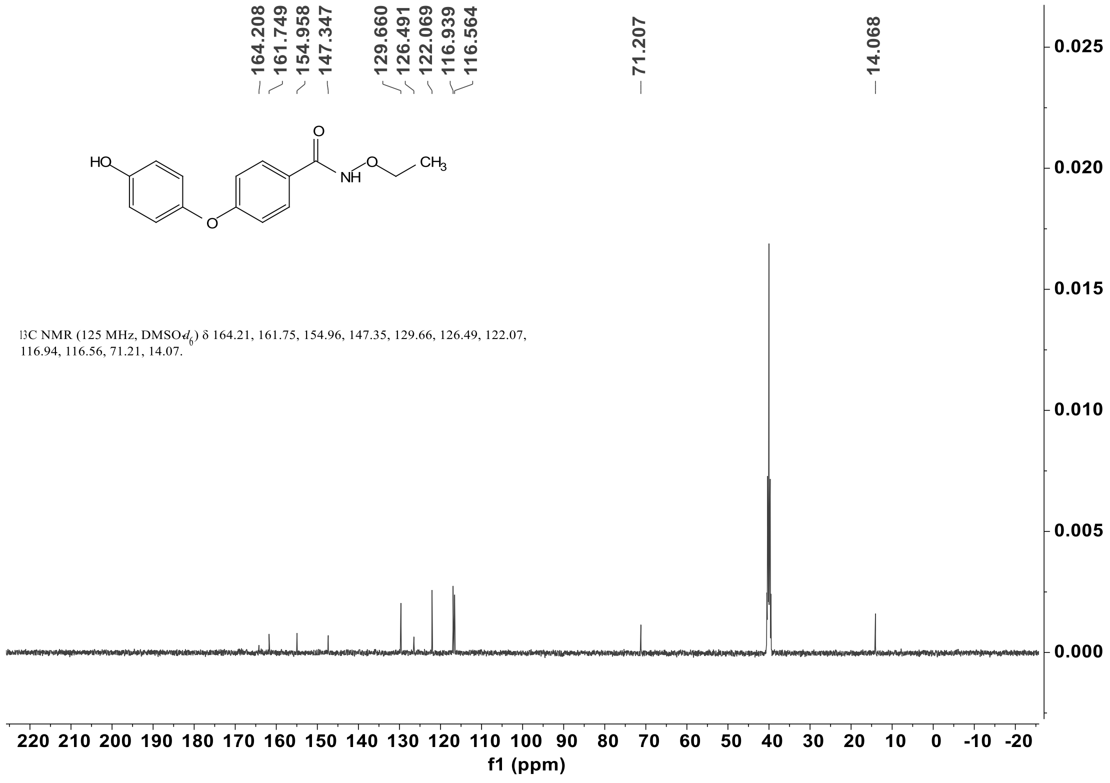

The ^{13}C NMR spectrogram of compound **M2**

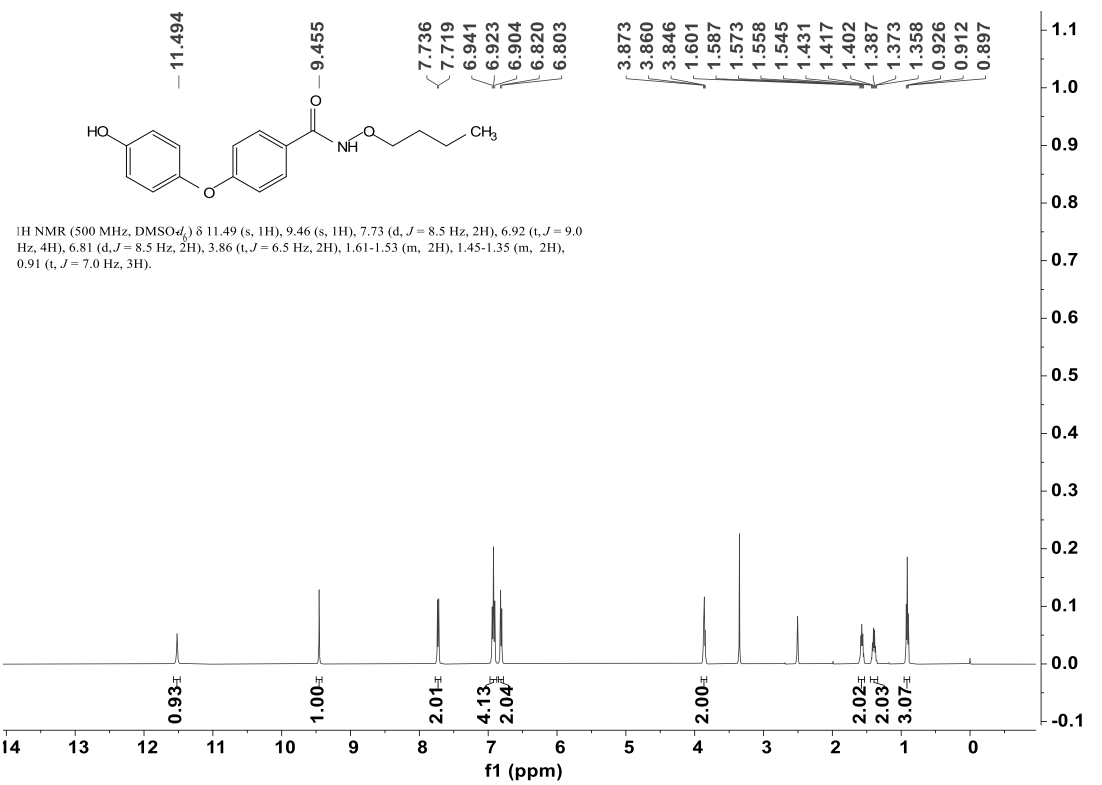

The ^{1}H NMR spectrogram of compound **M3**

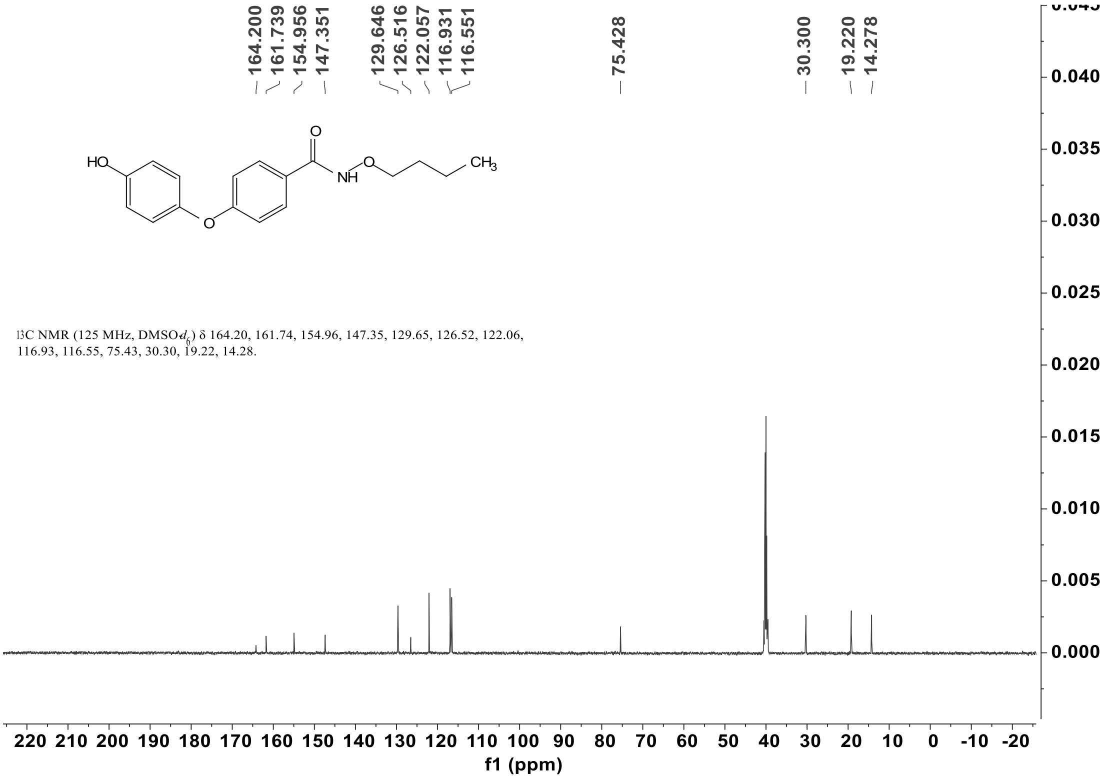

The ^{13}C NMR spectrogram of compound **M3**

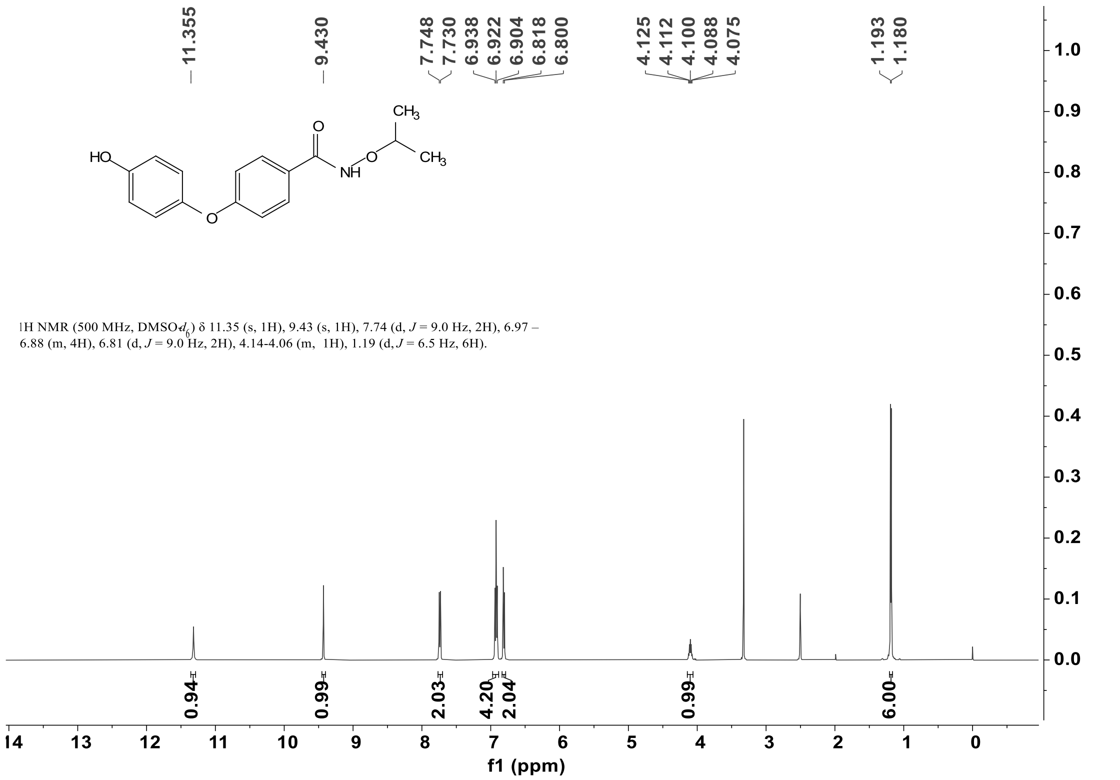

The ^{1}H NMR spectrogram of compound **M4**

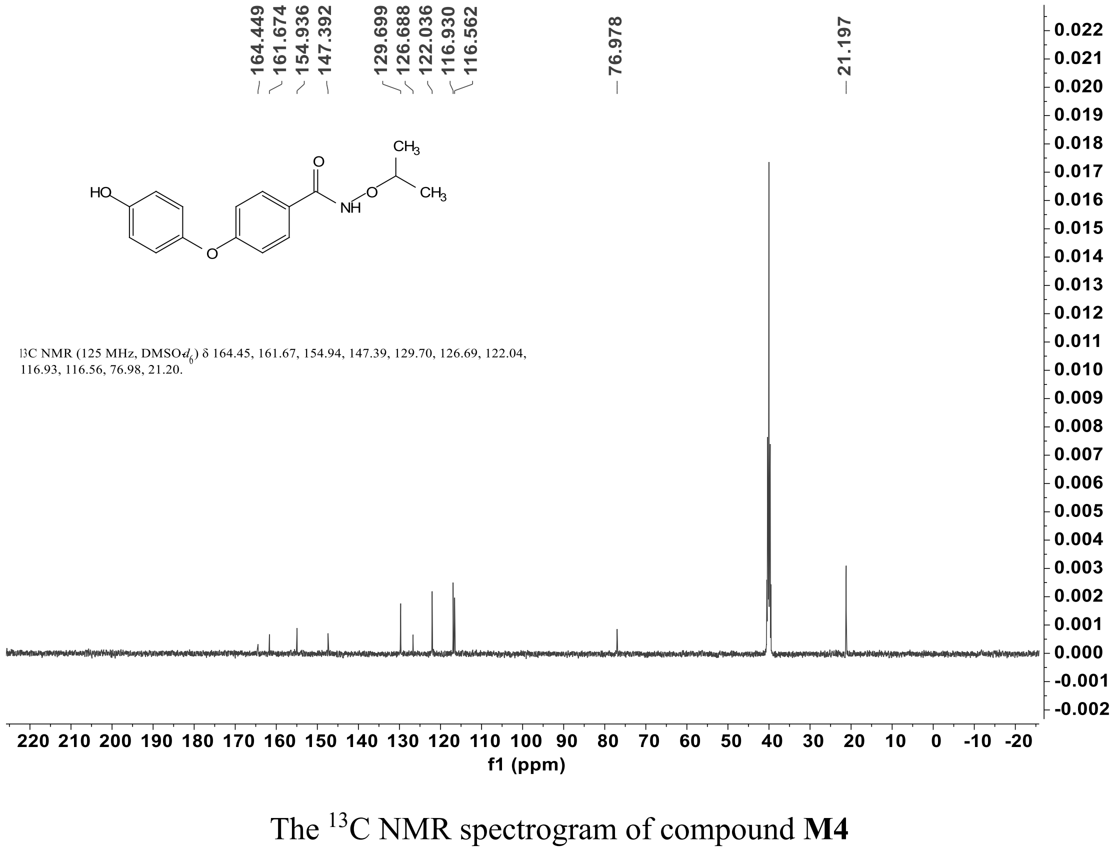

The ^{13}C NMR spectrogram of compound **M4**

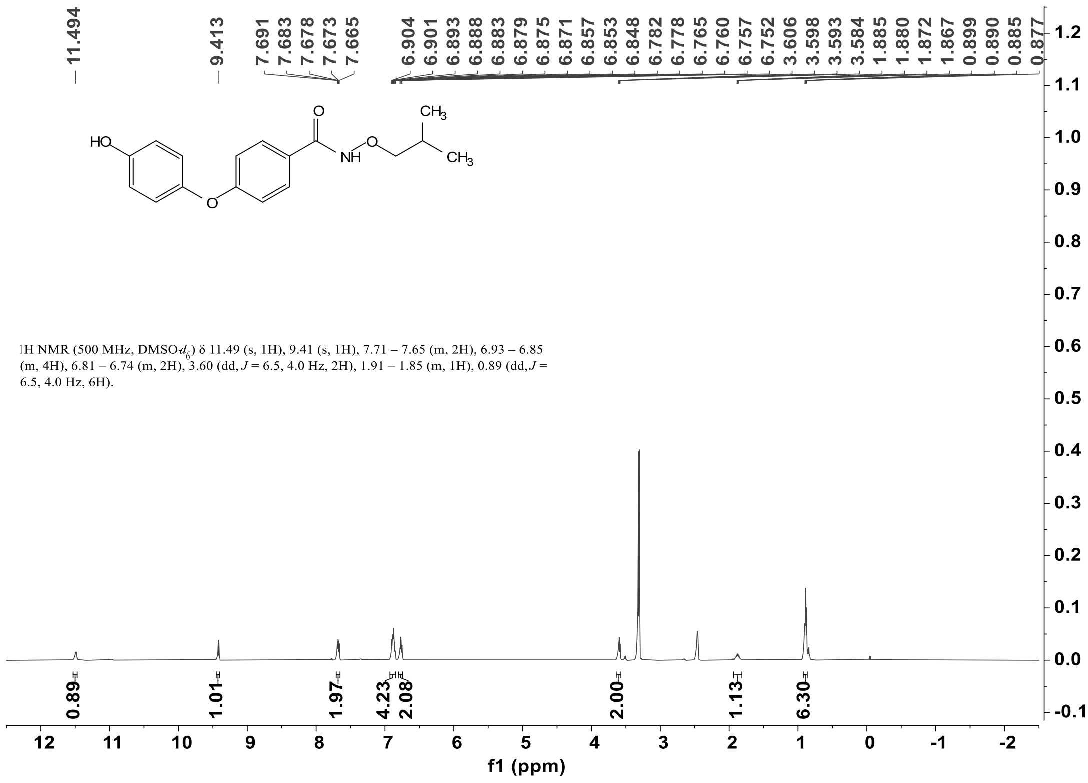

The ^{1}H NMR spectrogram of compound **M5**

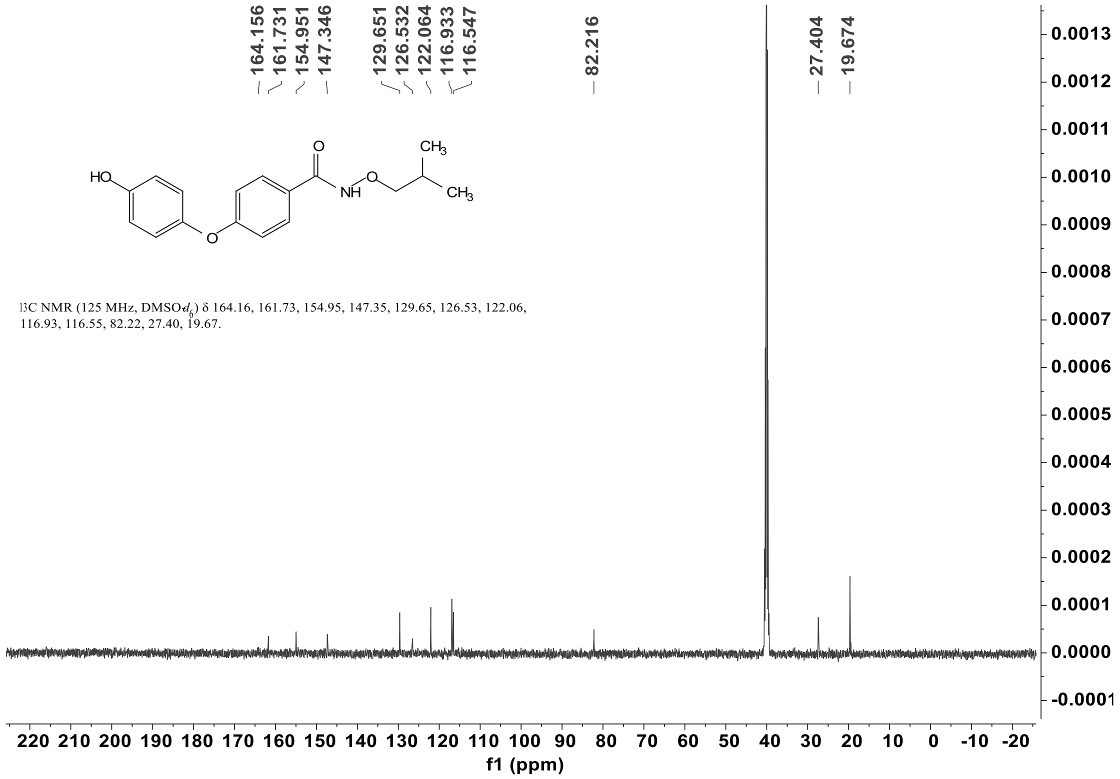

The ^{13}C NMR spectrogram of compound **M5**

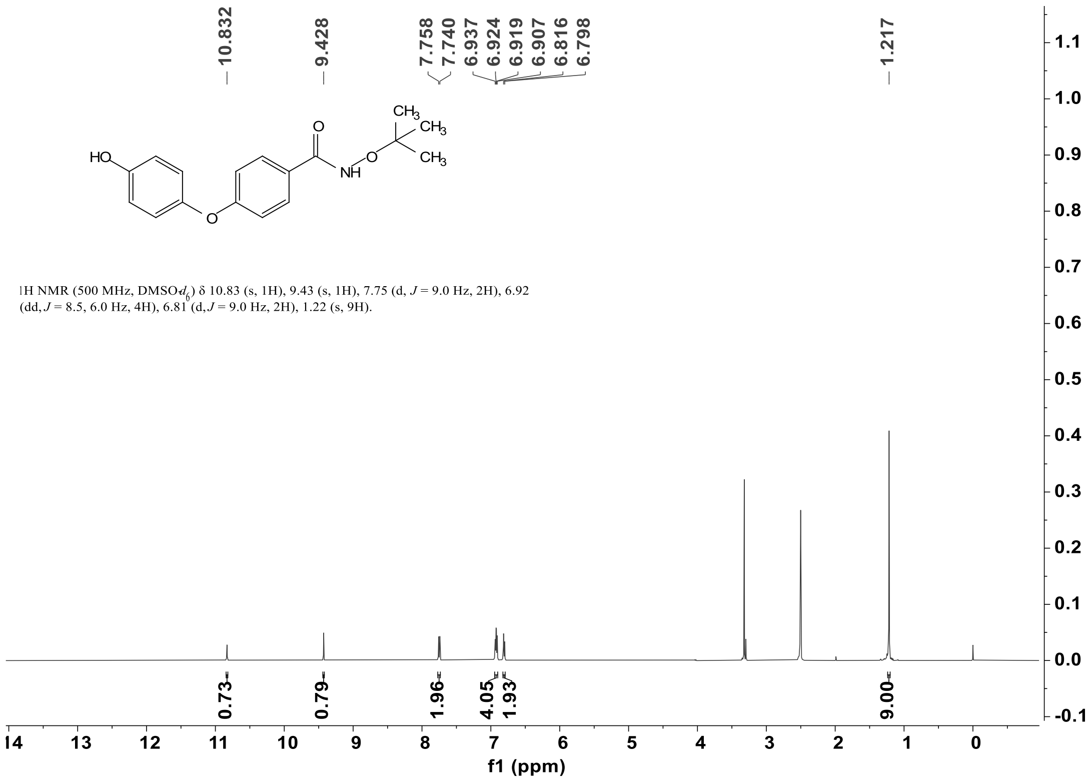

The ^{1}H NMR spectrogram of compound **M6**

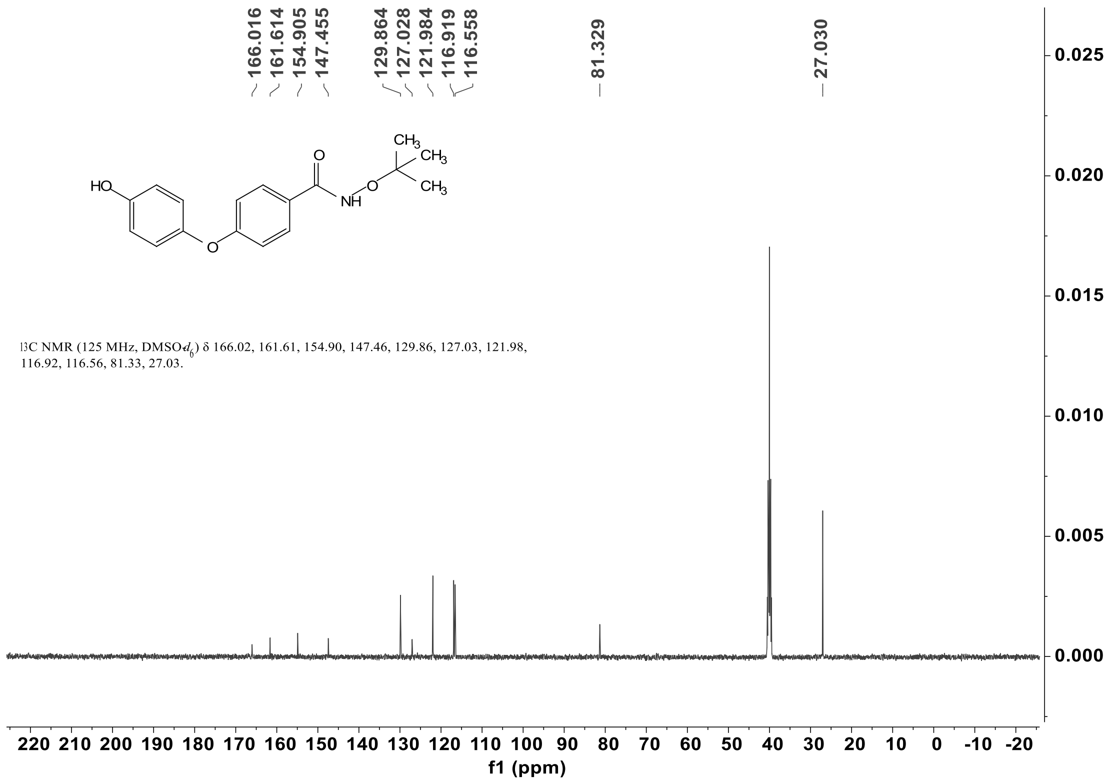

The ^{13}C NMR spectrogram of compound **M6**

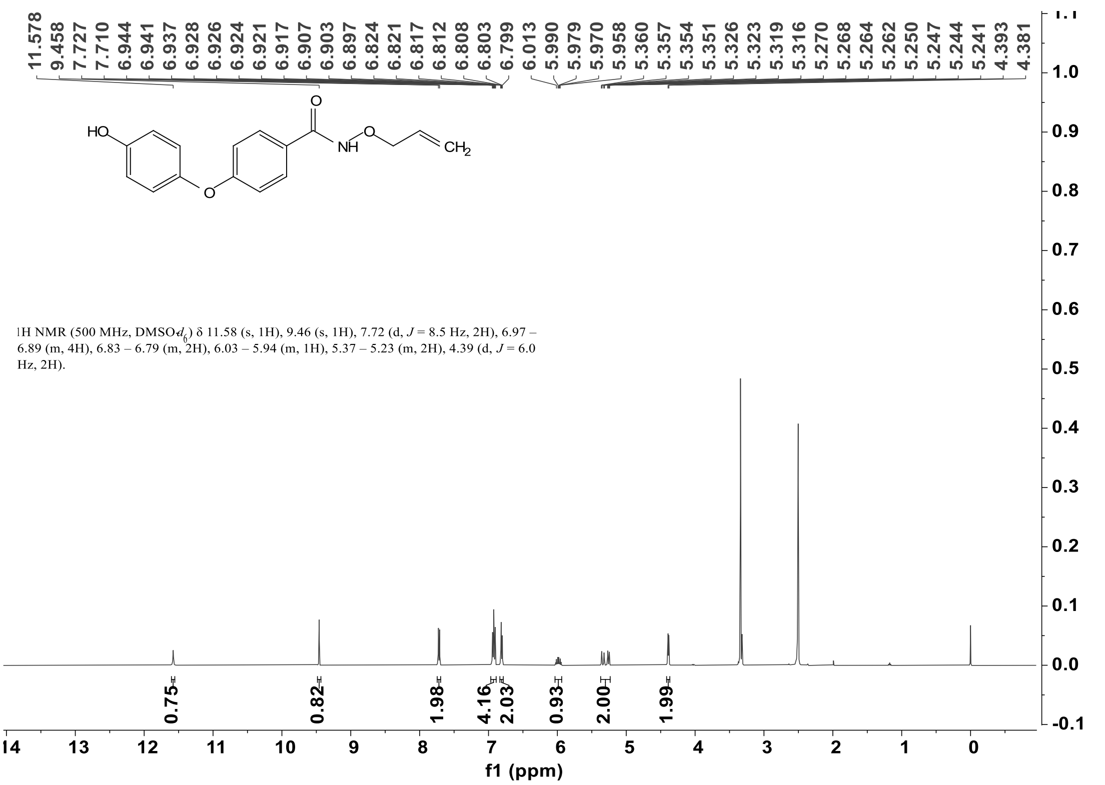

The ^{1}H NMR spectrogram of compound **M7**

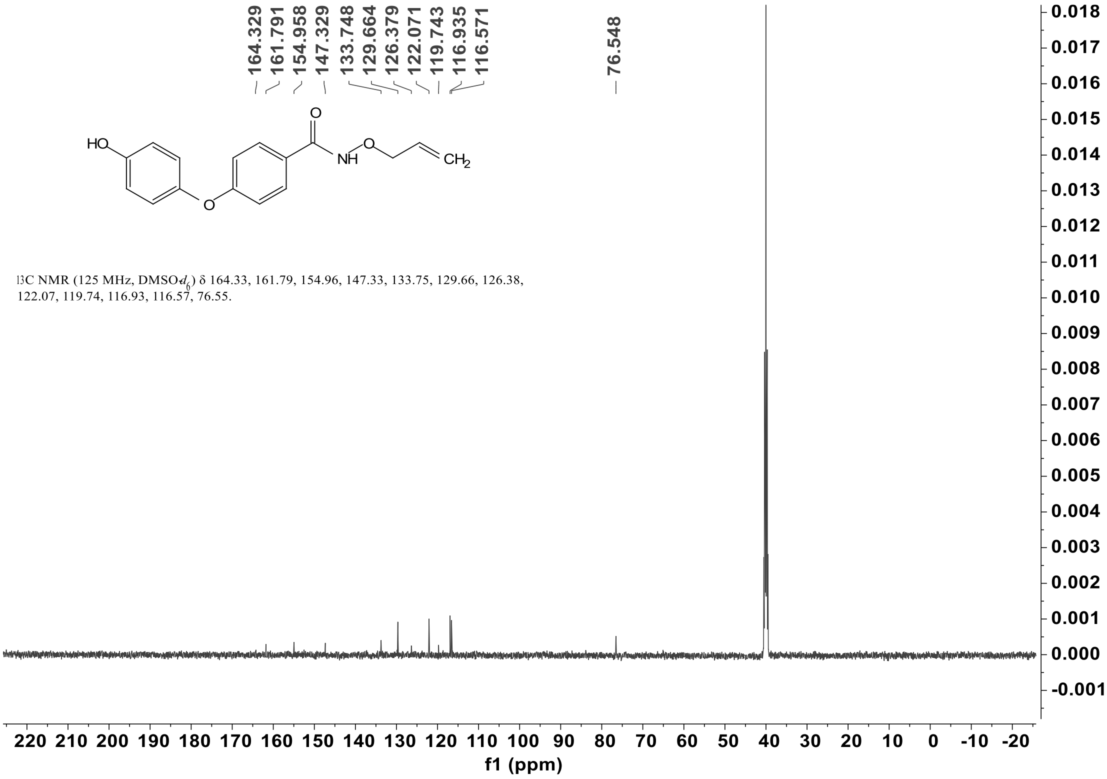

The ^{13}C NMR spectrogram of compound **M7**

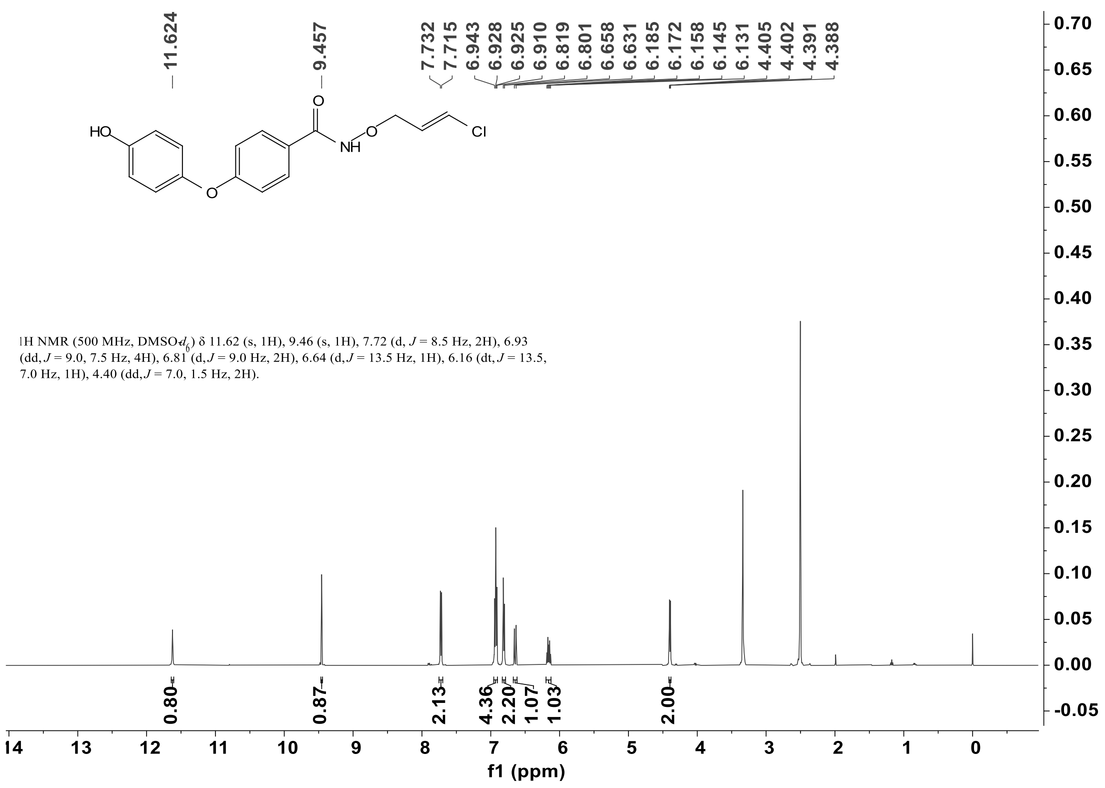

The ^{1}H NMR spectrogram of compound **M8**

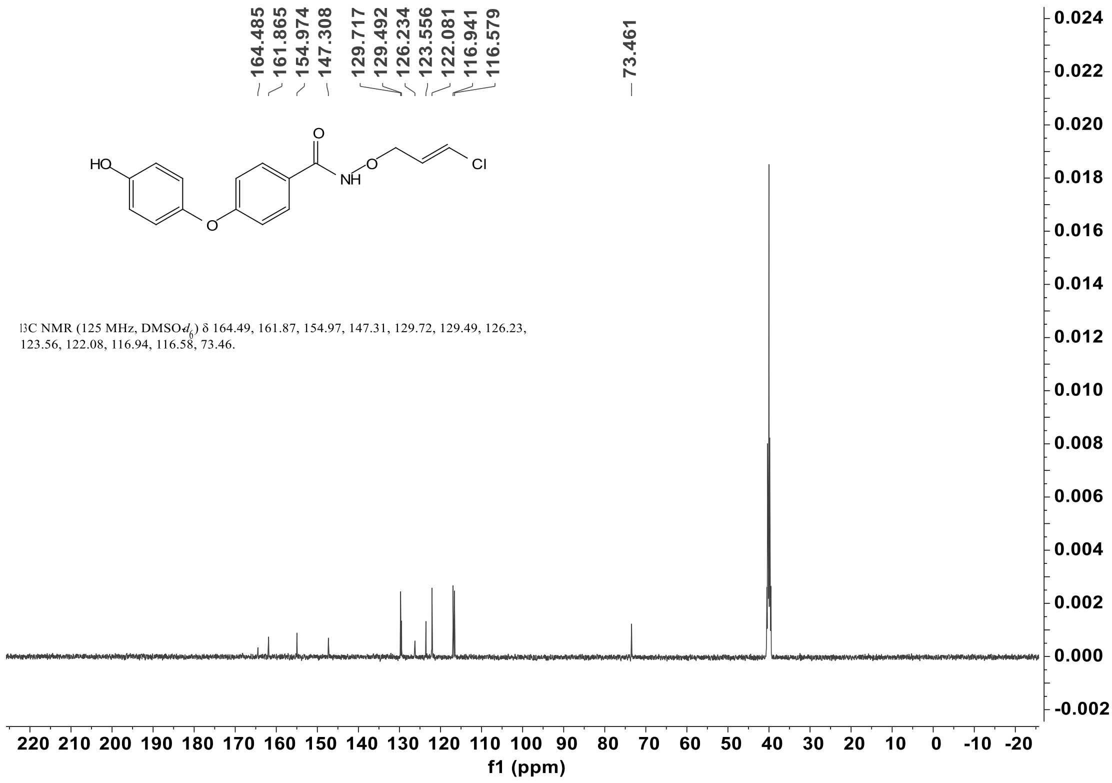

The ^{13}C NMR spectrogram of compound **M8**

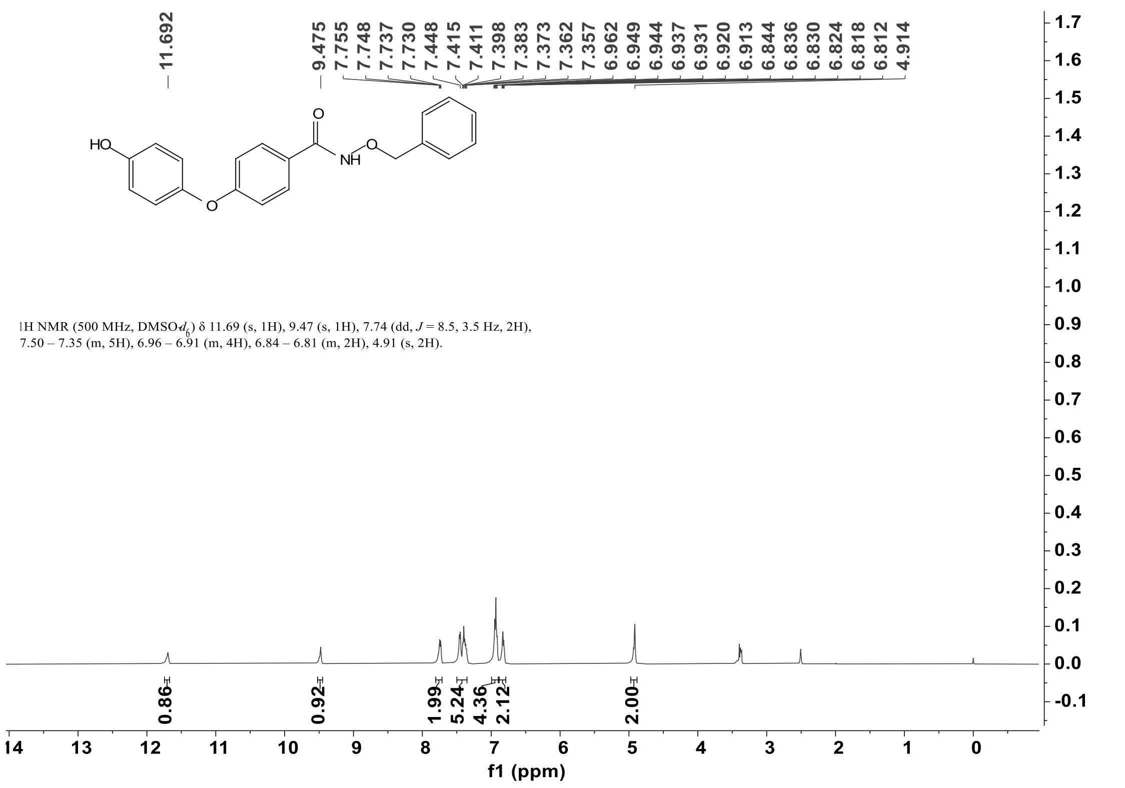

The ^{1}H NMR spectrogram of compound **M9**

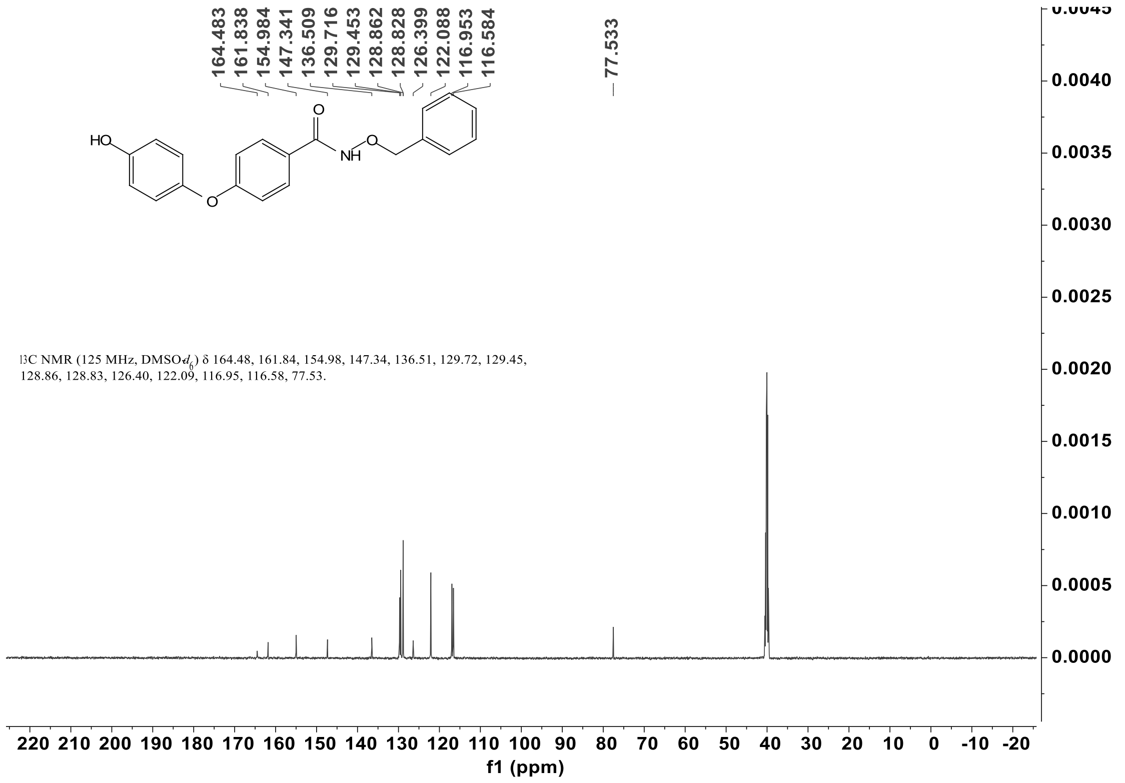

The ^{13}C NMR spectrogram of compound **M9**

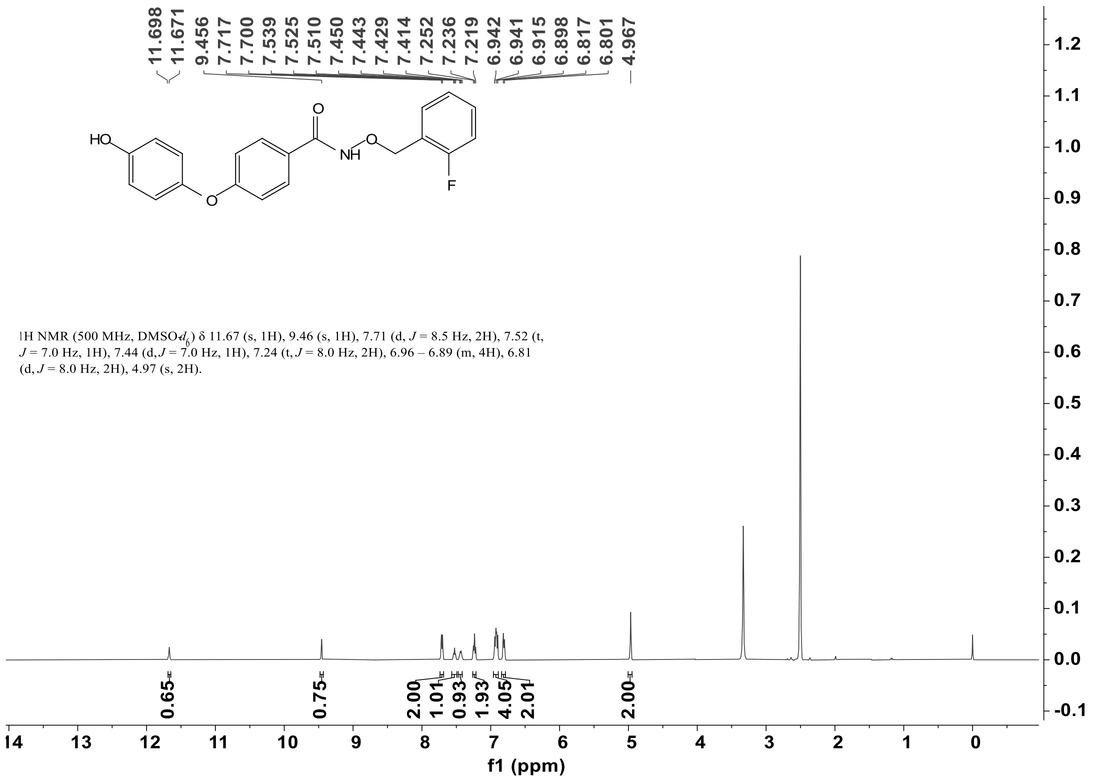

The ^{1}H NMR spectrogram of compound **M10**

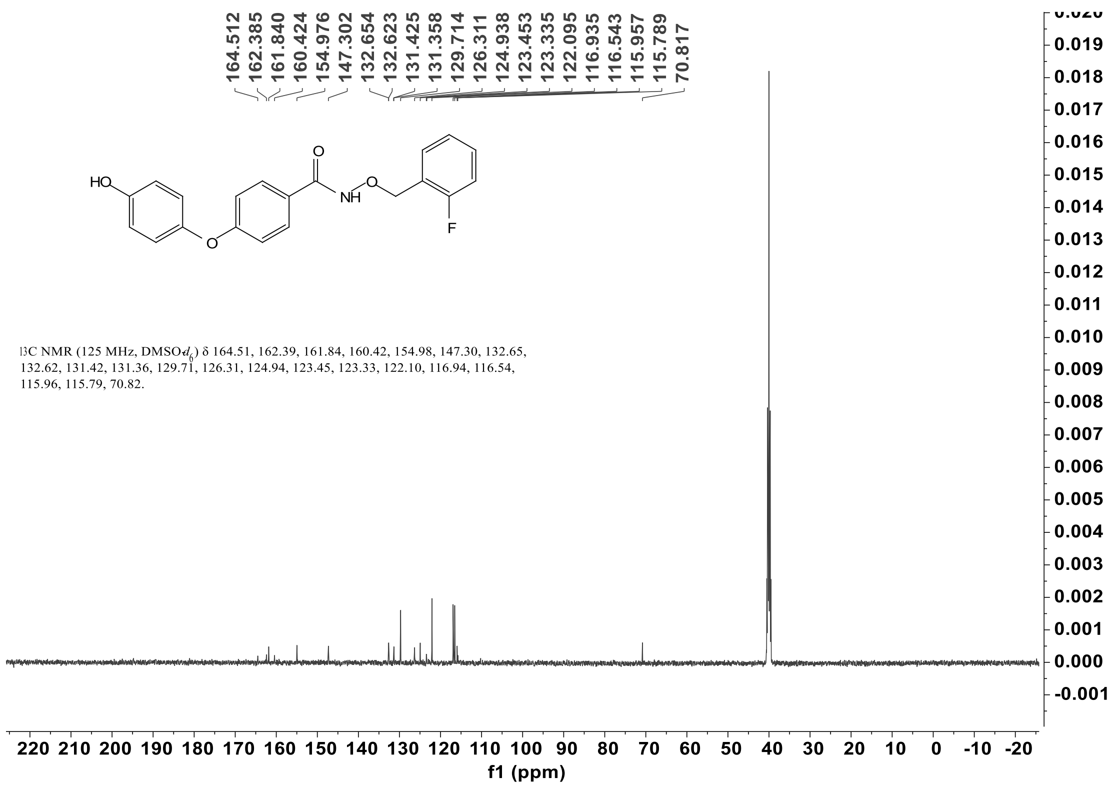

The ^{13}C NMR spectrogram of compound **M10**

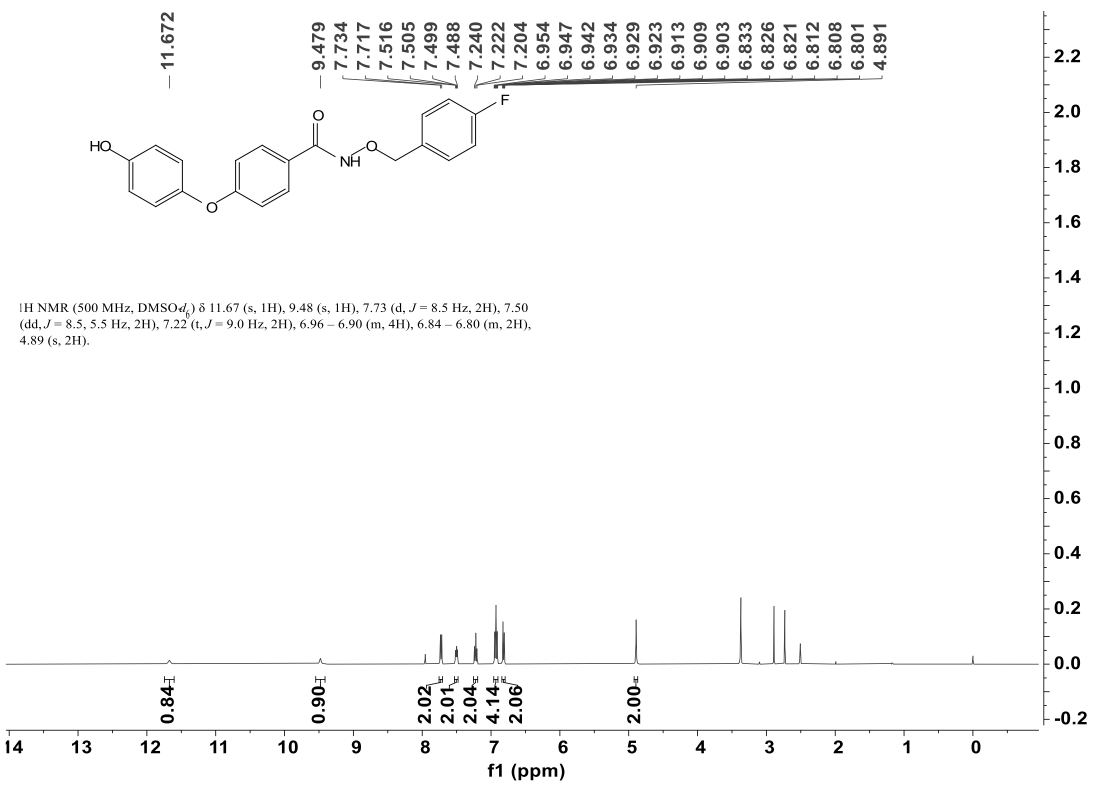

The ^{1}H NMR spectrogram of compound **M11**

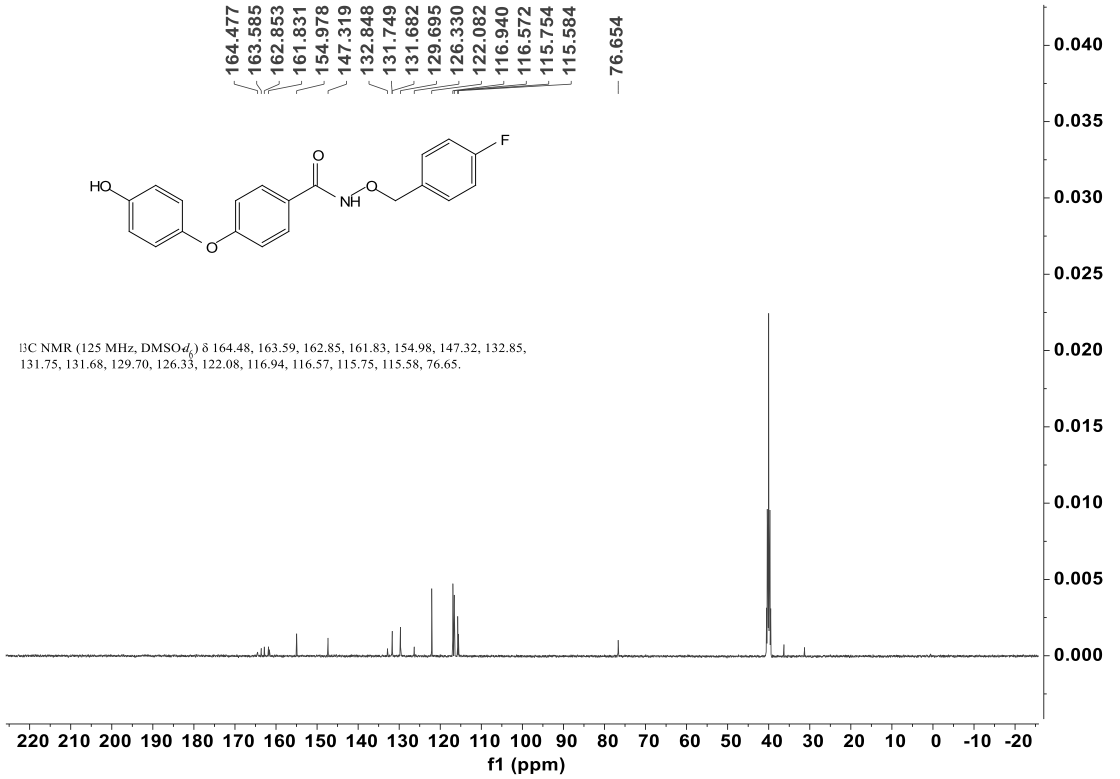

The ^{13}C NMR spectrogram of compound **M11**

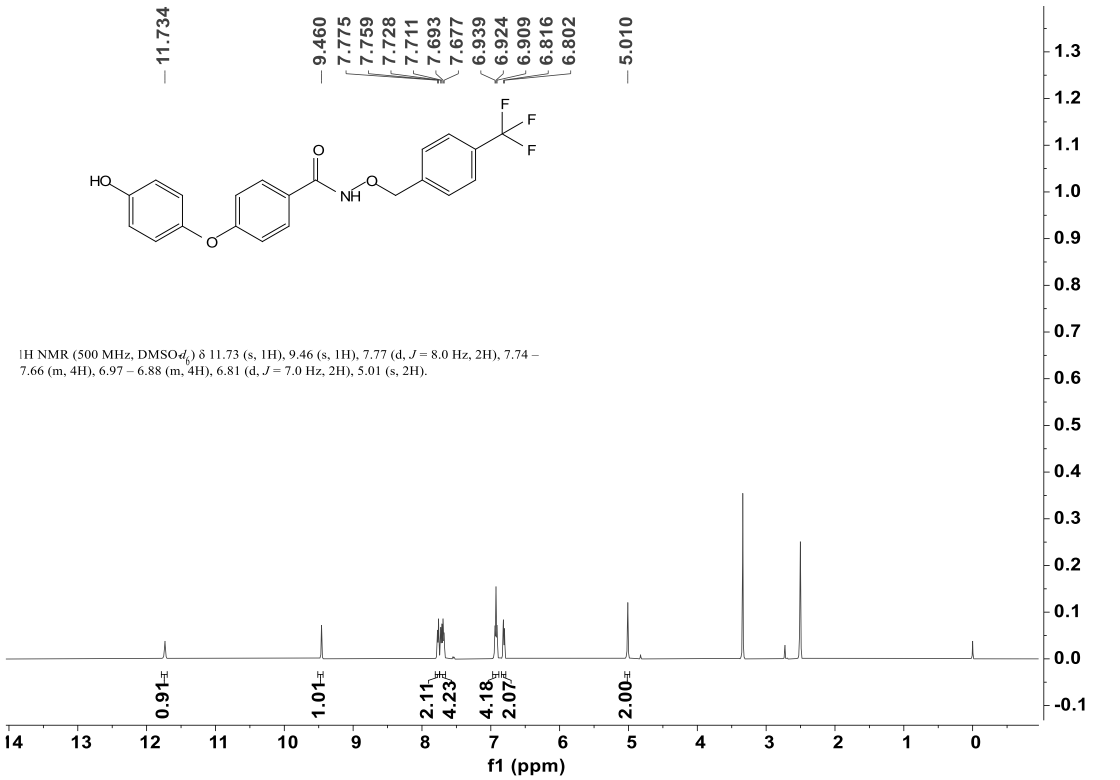

The ^{1}H NMR spectrogram of compound **M12**

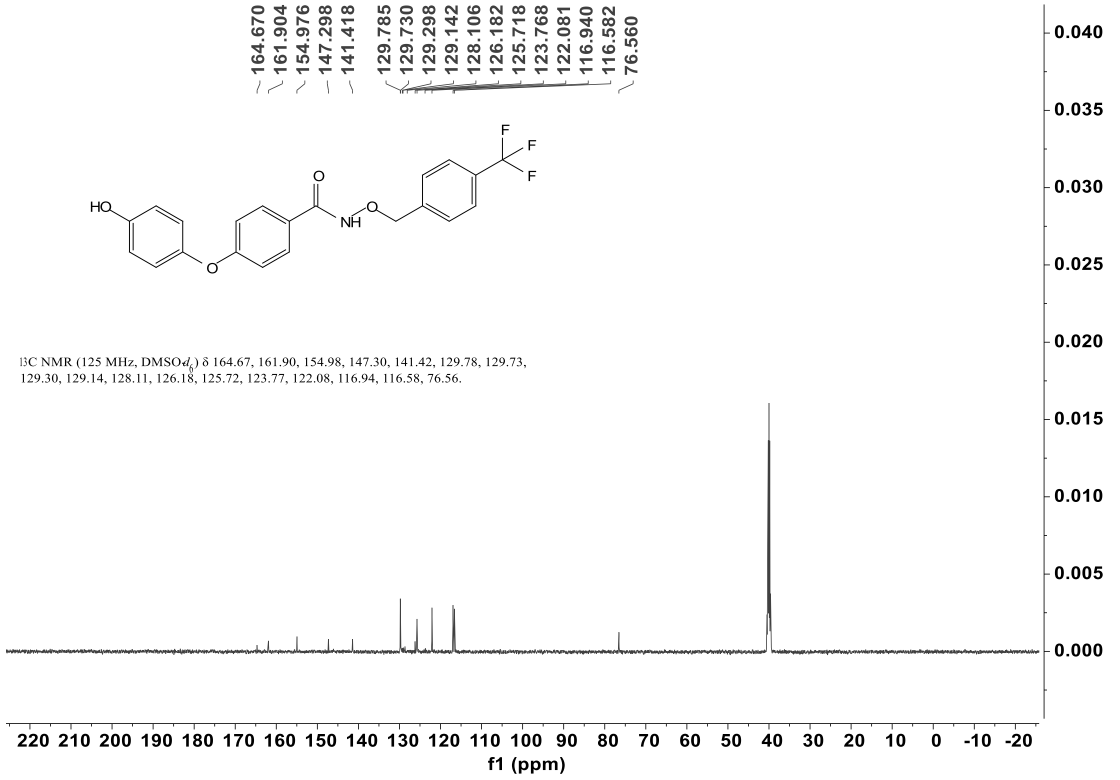

The ^{13}C NMR spectrogram of compound **M12**

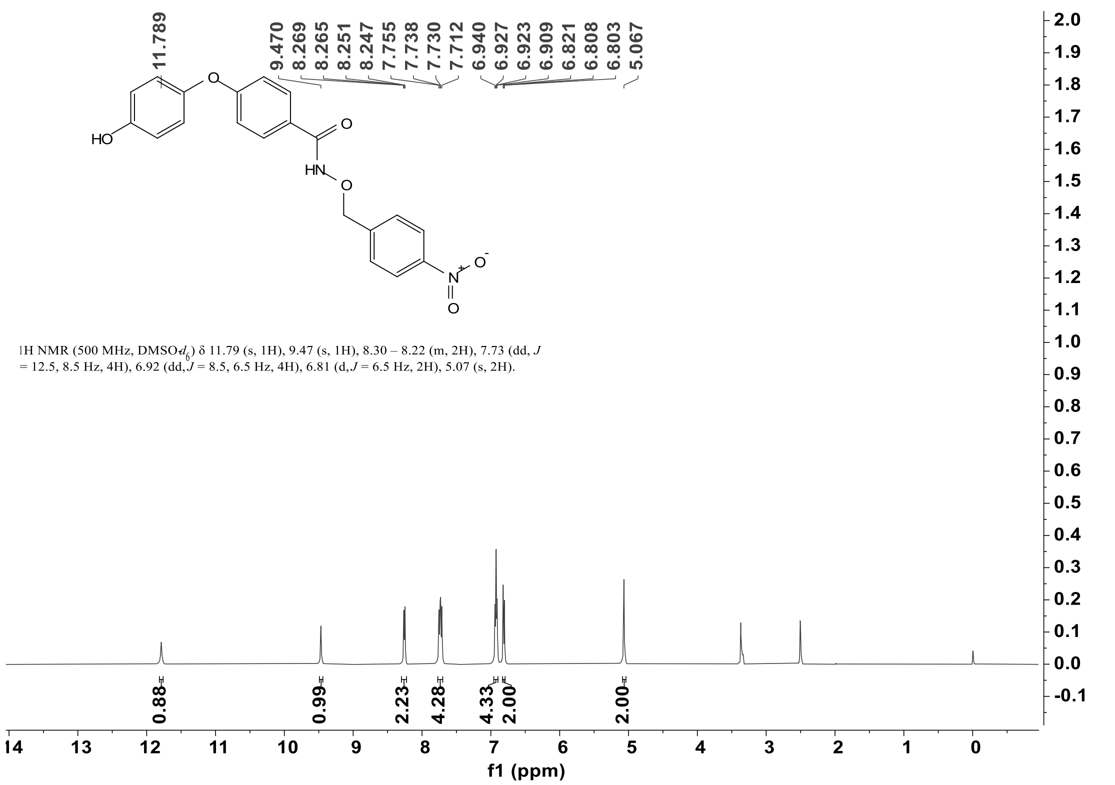

The ^{1}H NMR spectrogram of compound **M13**

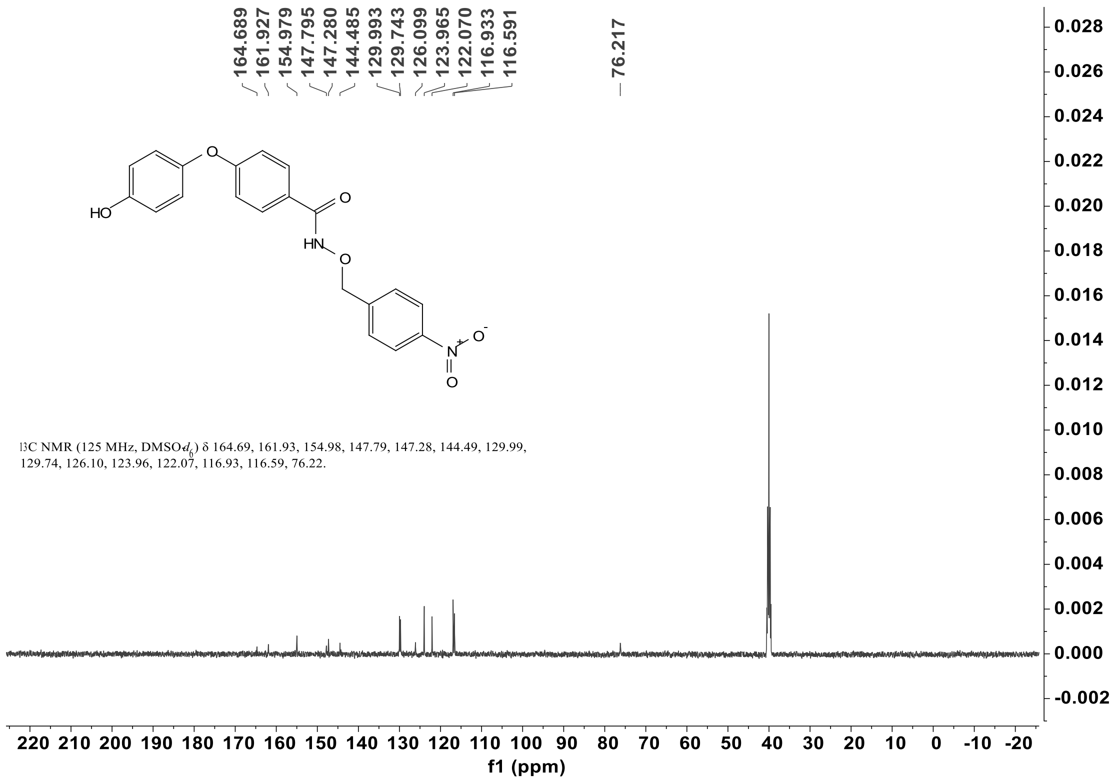

The ^{13}C NMR spectrogram of compound **M13**

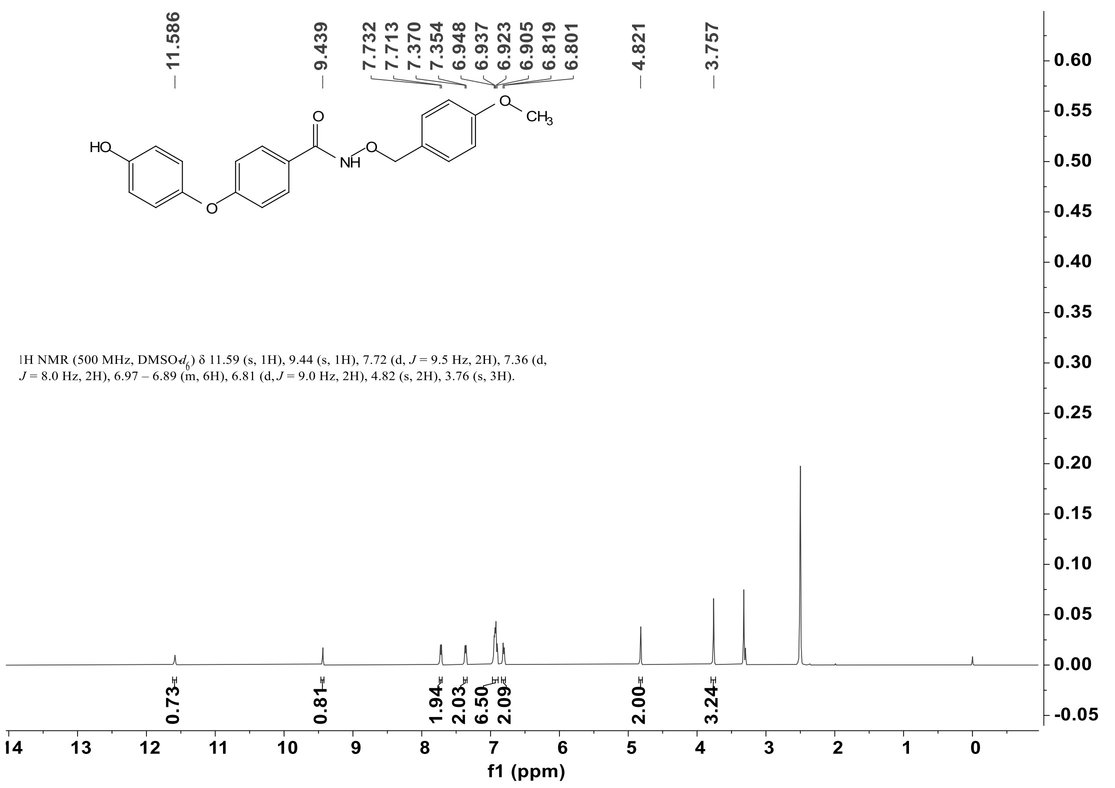

The ^{1}H NMR spectrogram of compound **M14**

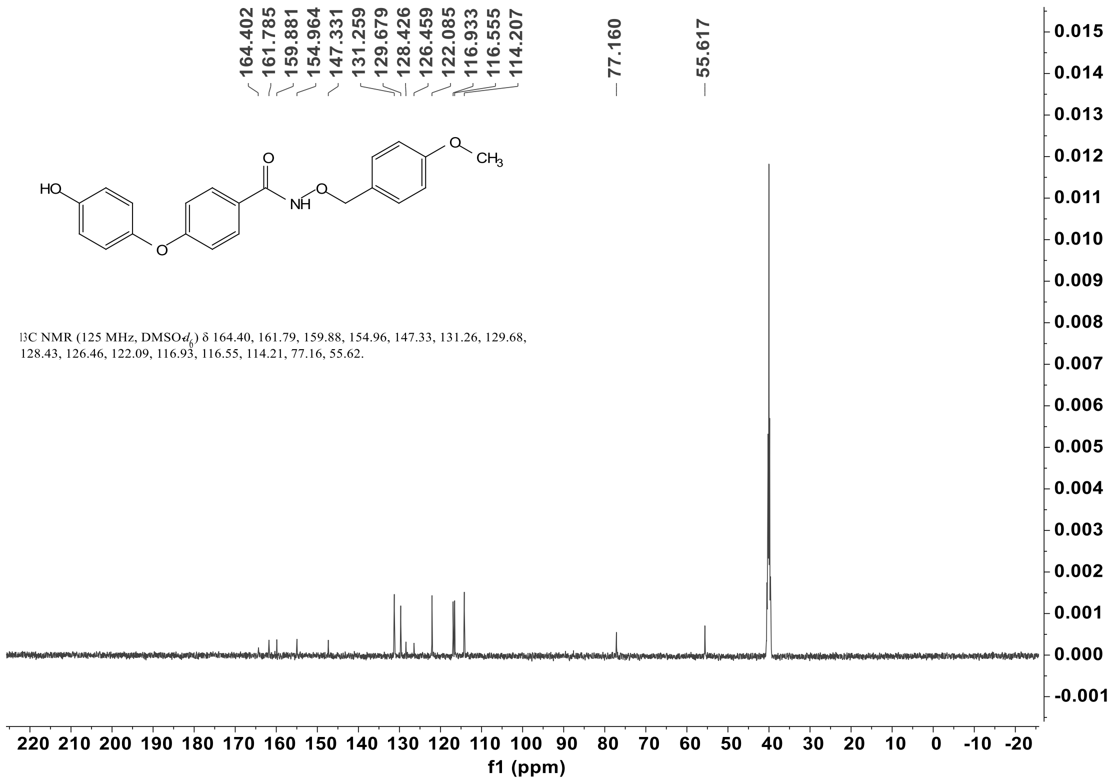

The ^{13}C NMR spectrogram of compound **M14**

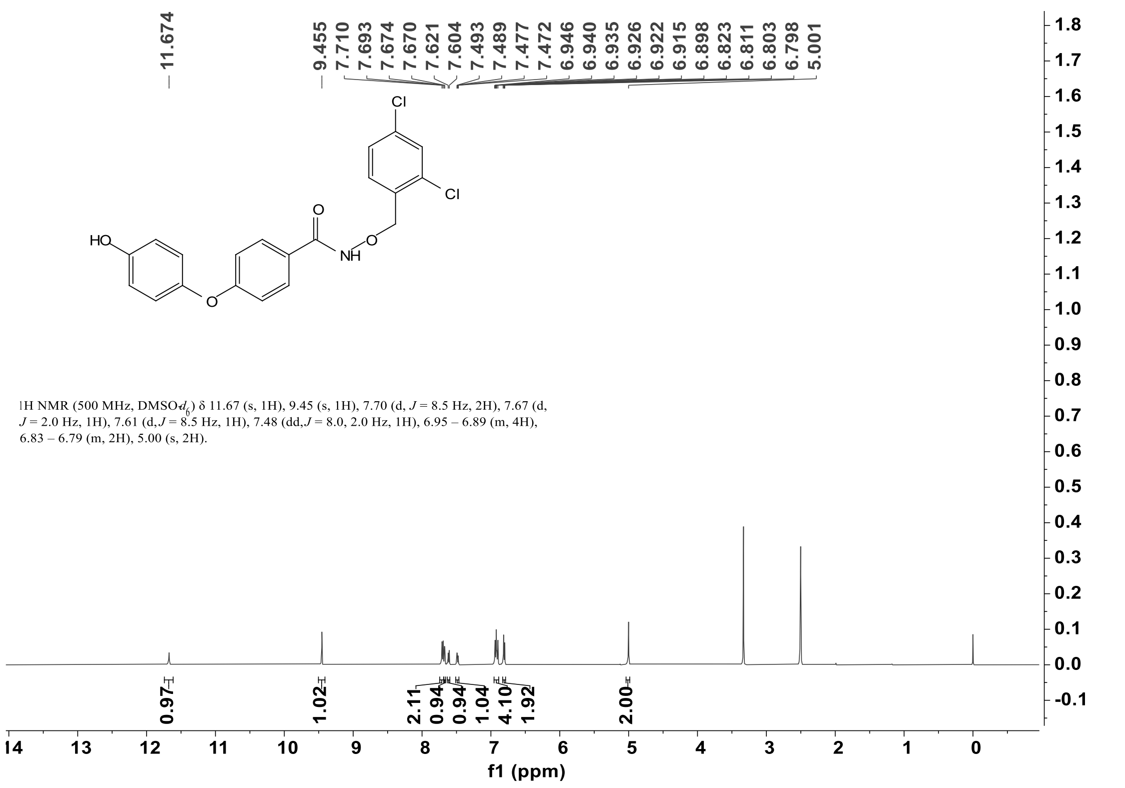

The ^{1}H NMR spectrogram of compound **M15**

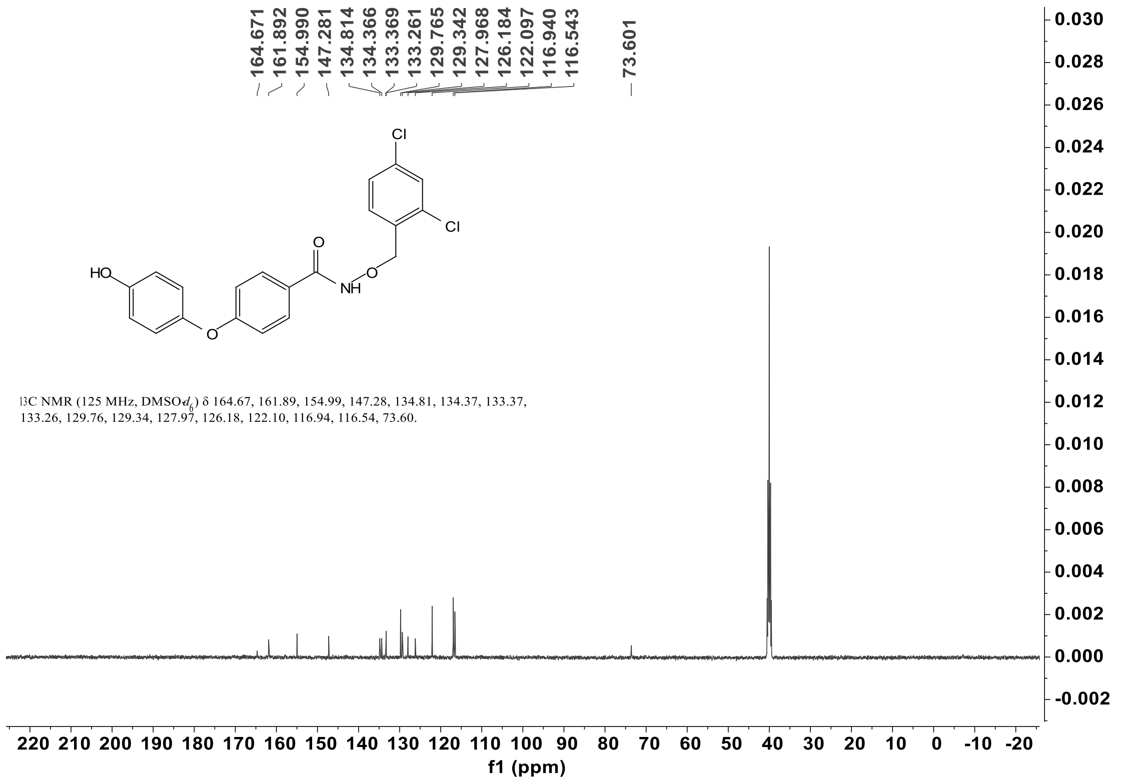

The ^{13}C NMR spectrogram of compound **M15**

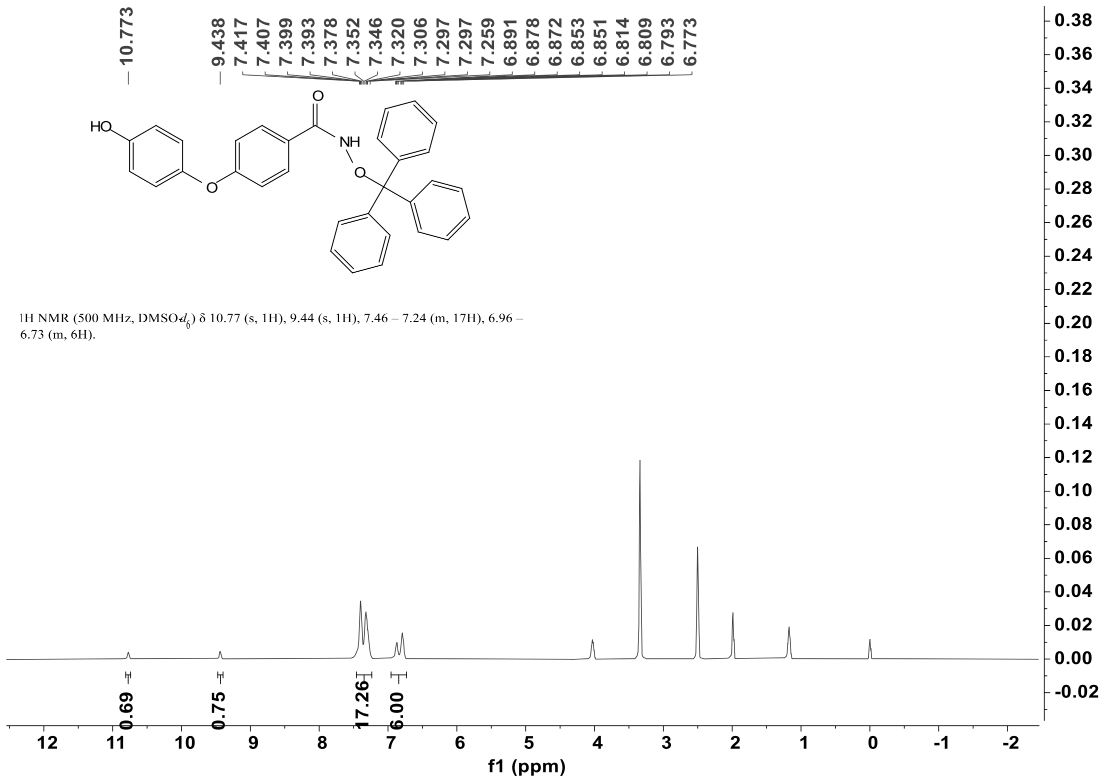

The ^{1}H NMR spectrogram of compound **M16**

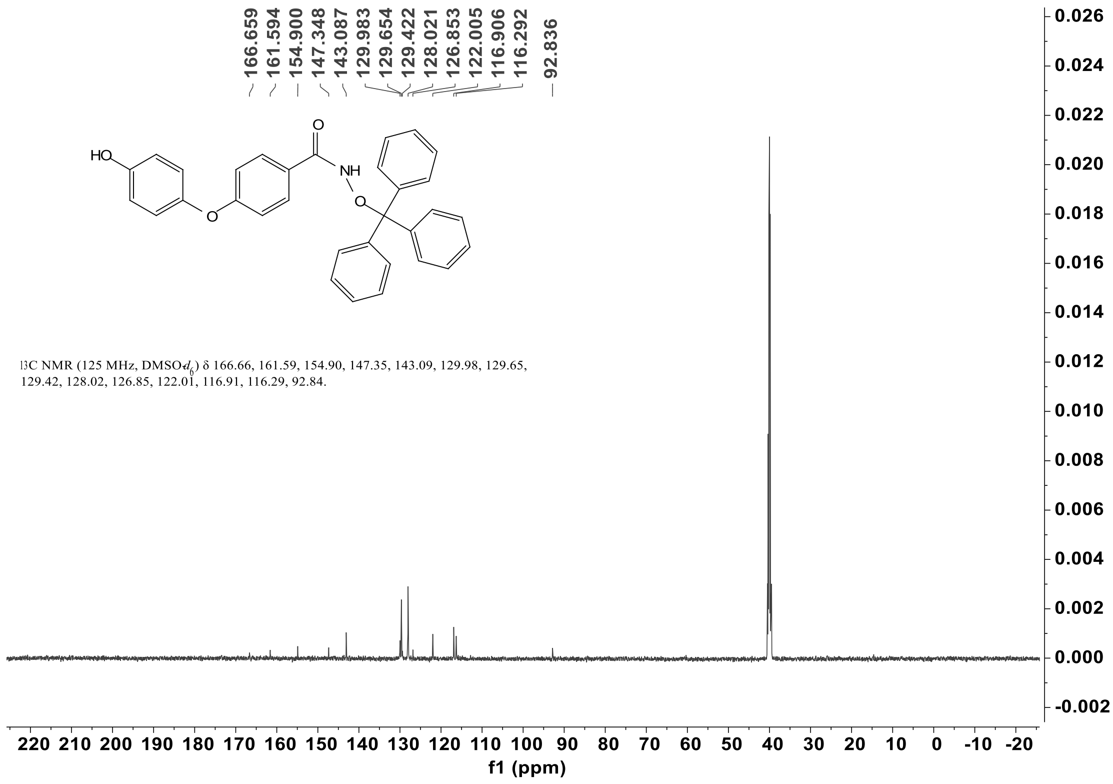

The ^{13}C NMR spectrogram of compound **M16**

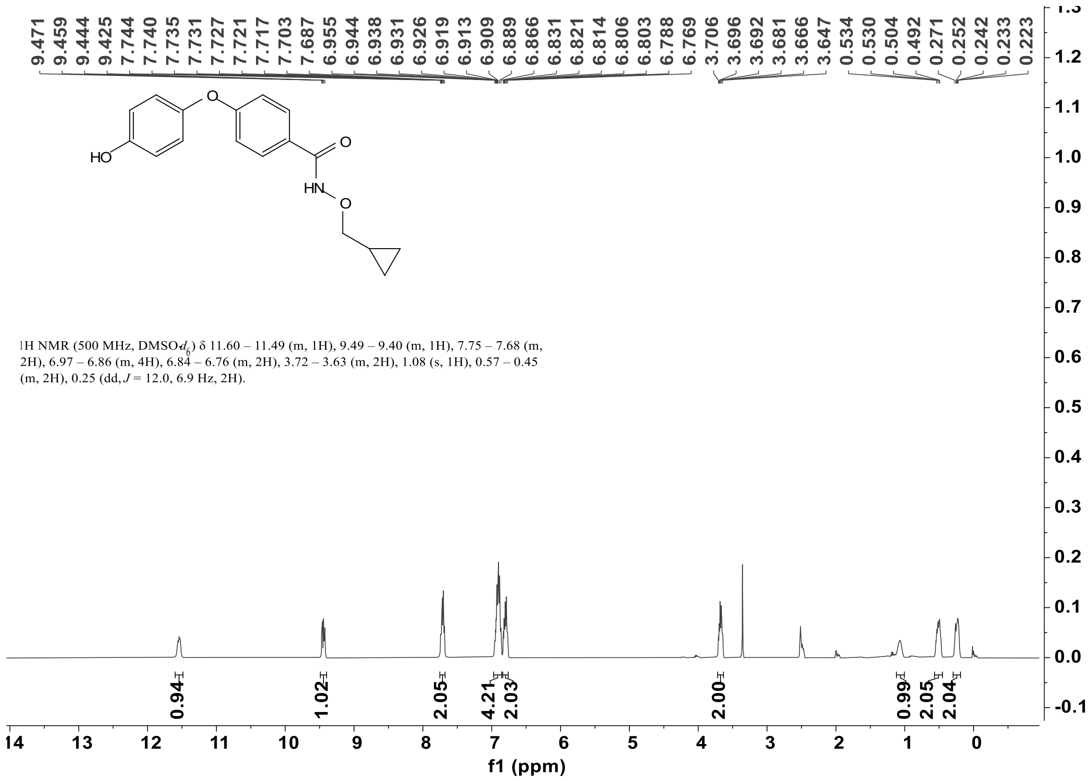

The ^{1}H NMR spectrogram of compound **M17**

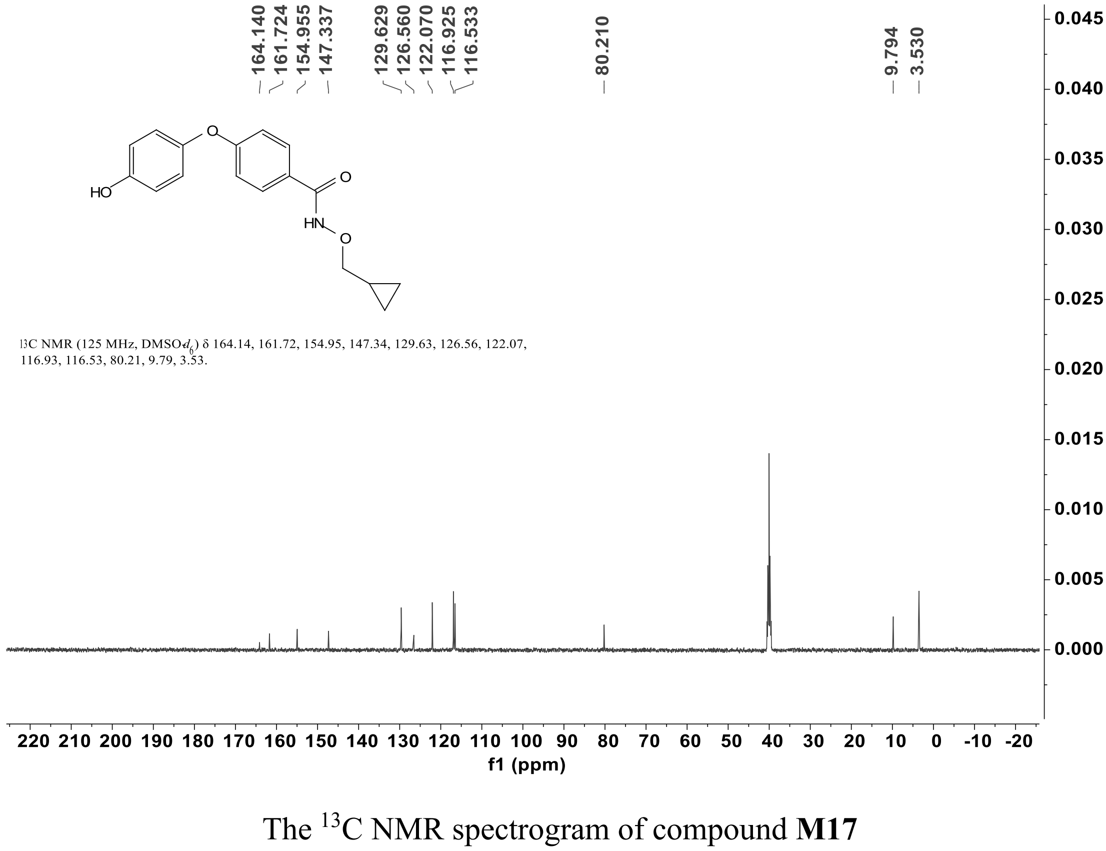

The ^{13}C NMR spectrogram of compound **M17**

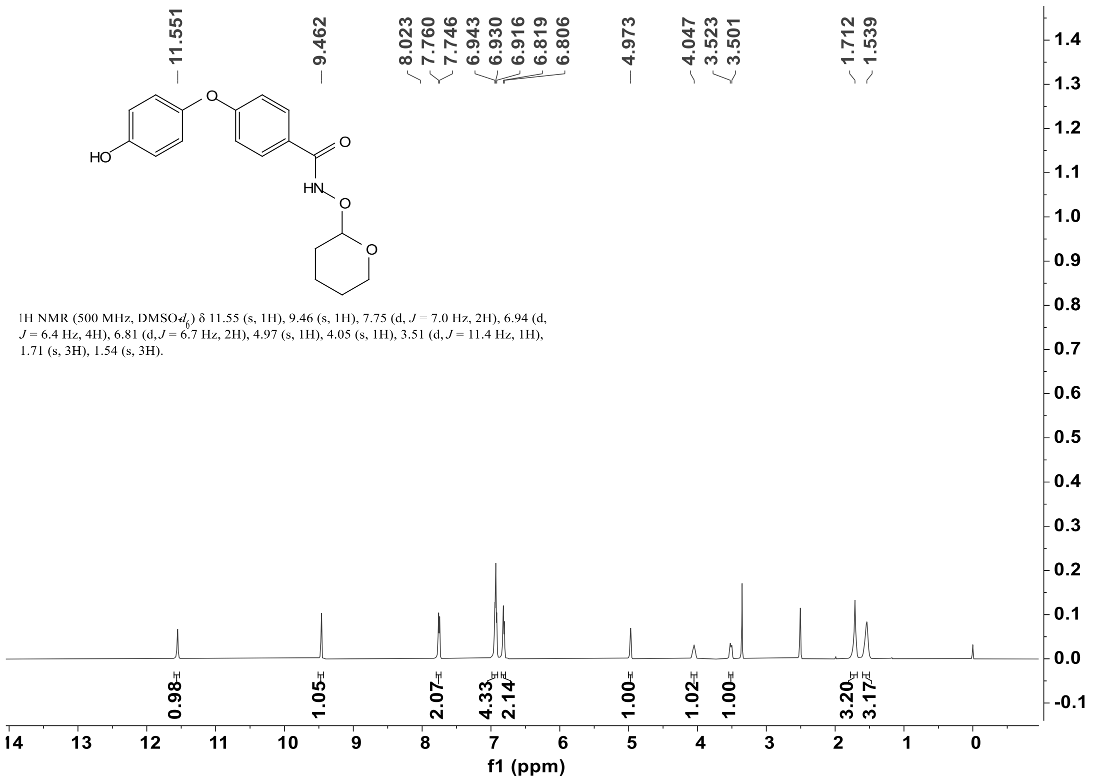

The ^{1}H NMR spectrogram of compound **M18**

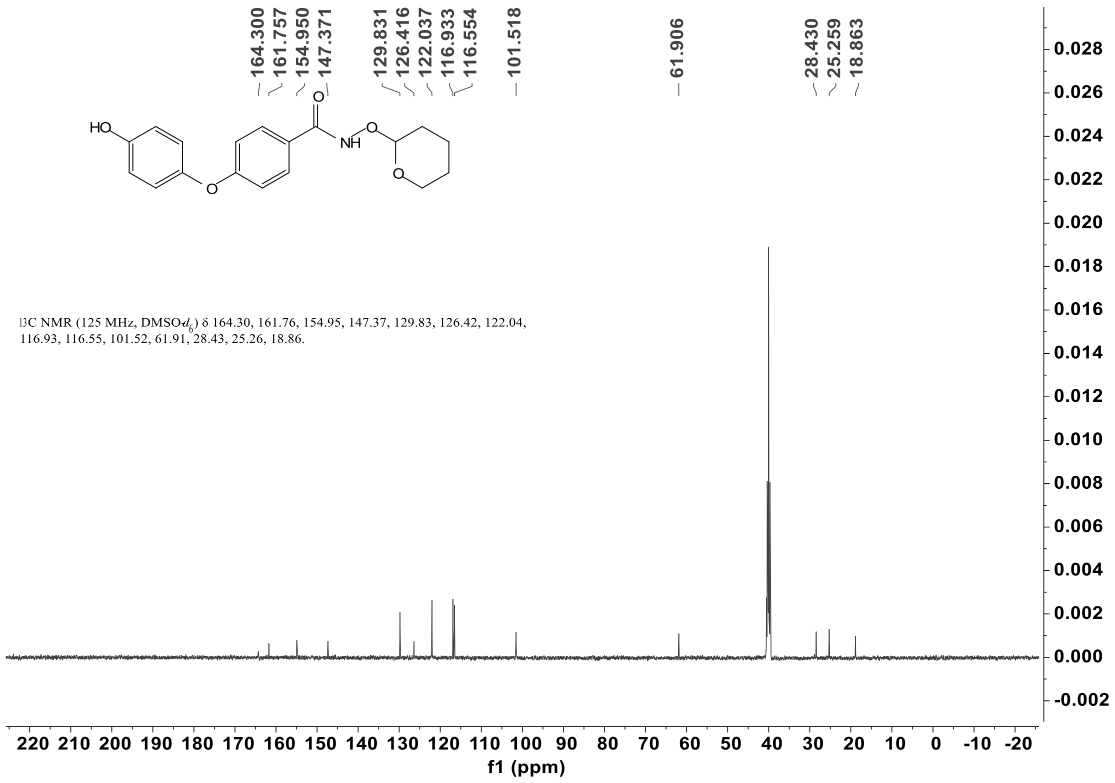

The ^{13}C NMR spectrogram of compound **M18**
